# Mechanistic In Situ and Ex Situ Studies of Phase Transformations in Molecular Co‐Crystals

**DOI:** 10.1002/chem.202002267

**Published:** 2020-10-07

**Authors:** Alexander E. Clout, Asma B. M. Buanz, Yuying Pang, Wing‐Mei Tsui, Dongpeng Yan, Gary Parkinson, Timothy J. Prior, Dejan‐Krešimir Bučar, Simon Gaisford, Gareth R. Williams

**Affiliations:** ^1^ UCL School of Pharmacy University College London 29-39 Brunswick Square London WC1N 1AX UK; ^2^ College of Chemistry Beijing Normal University Beijing 100875 China; ^3^ Department of Chemistry and Biochemistry University of Hull Hull HU6 7RX UK; ^4^ Department of Chemistry University College London 20 Gordon Street London WC1H 0AJ UK

**Keywords:** differential scanning calorimetry, hyphenated techniques, pharmaceutical co-crystals, synchrotron X-ray diffraction

## Abstract

Co‐crystallisation is widely explored as a route to improve the physical properties of pharmaceutical active ingredients, but little is known about the fundamental mechanisms of the process. Herein, we apply a hyphenated differential scanning calorimetry—X‐ray diffraction technique to mimic the commercial hot melt extrusion process, and explore the heat‐induced synthesis of a series of new co‐crystals containing isonicotinamide. These comprise a 1:1 co‐crystal with 4‐hydroxybenzoic acid, 2:1 and 1:2 systems with 4‐hydroxyphenylacetic acid and a 1:1 crystal with 3,4‐dihydroxyphenylactic acid. The formation of co‐crystals during heating is complex mechanistically. In addition to co‐crystallisation, conversions between polymorphs of the co‐former starting materials and co‐crystal products are also observed. A subsequent study exploring the use of inkjet printing and milling to generate co‐crystals revealed that the synthetic approach has a major effect on the co‐crystal species and polymorphs produced.

## Graphical abstract

**Co‐crystallisation** is widely explored as a route to improve the physical properties of pharmaceutical active ingredients, but little is known about the fundamental mechanisms of the process. In this study, X‐ray diffraction was used to provide an unprecedented insight into pharmaceutical co‐crystallisation processes (see figure).

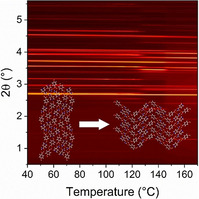

## Introduction

Co‐crystallisation is a method by which the physical properties of a molecule can be altered without making or breaking any covalent bonds.^[1]^ This can directly affect the properties of an active pharmaceutical ingredient (API), and (for instance) may improve its solubility and/or rate of dissolution. As a consequence of the substance existing in a crystalline form, co‐crystals are likely to be more stable, reproducible and easier to purify than other solid forms of a drug, and, therefore, are more desirable.^[1]^ A number of approaches exist to identify suitable co‐formers for an API,[^2^, ^3^, ^4^] but at the moment there is little understanding of the mechanism by which the crystals form. To explore this, non‐invasive probes are required. While these are easy to implement in the liquid or gas phase, non‐invasive probes for the solid state are lacking.

Differential scanning calorimetry (DSC) is the main method used in the pharmaceutical sciences to study how the physical state of materials changes as a function of temperature. However, it does not allow definitive structural elucidation. Powder X‐ray diffraction (XRD) is the “gold standard” analytical technique for the identification of crystalline forms, but standard powder diffractometers are unable to heat the sample sufficiently rapidly to mirror DSC heating rates (ca. 10 °C min^−1^), and typically require of the order of 10–30 min to collect a high‐quality diffraction pattern. Sequential analysis is possible, whereby a material could be heated in a DSC (or in an oven if larger masses are required) and subsequently analysed by XRD to determine the physical form. However, the physical form may change as the sample cools, particularly if metastable materials are generated. To overcome this issue, we recently developed a new hyphenated DSC‐XRD analytical method.^[5]^ This approach has led to enhanced understanding of phase transitions in glutaric acid and sulfathiazole,^[5]^ carbamazepine and dihydrocarbamazapine,^[6]^ and paracetamol.^[7]^ Most recently, we employed the DSC‐XRD platform with crystal structure prediction work and were able to identify and solve the structure of a new polymorph of olanzapine.^[8]^

Herein, we extend the DSC‐XRD approach to explore the fabrication of co‐crystals by thermal methods. Understanding these processes is important, because for industrial applications co‐crystals are most likely to be prepared using hot melt extrusion (HME), a continuous manufacturing approach which applies heat energy to an intimate mixture of the co‐formers to generate a co‐crystal.[^4^, ^9^, ^10^] The DSC‐XRD approach will permit us to understand the transitions occurring during the thermal synthesis of co‐crystals, and as a result to design suitable HME manufacturing protocols.

**Figure 1:**
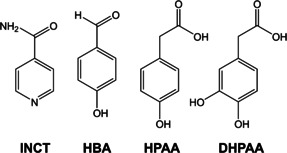
Structures of INCT and the co‐formers employed in this work.

As proof‐of‐concept we have developed four new co‐crystals based on isonicotinamide (INCT, Figure 1). INCT has been used extensively as a pharmaceutical co‐former to improve the solubility of an API without compromising its efficacy or stability.[^11^, ^12^, ^13^, ^14^, ^15^, ^16^, ^17^, ^18^, ^19^, ^20^, ^21^, ^22^] INCT is able to act as a hydrogen bond acceptor through the pyridine group, and the amide moiety is capable of engaging in a wide range of different hydrogen‐bonding motifs. Vishweshwar et al. generated a 1:1 co‐crystal^[23]^ between the antimicrobial and antioxidant^[24]^ API 4‐hydroxybenzoic acid (HBA, Figure 1) and INCT by crystallisation from hot methanol. In this work, we additionally explored 4‐hydroxyphenylacetic acid (HPAA, Figure 1) and 3,4‐dihydroxyphenylactic acid (DHPAA, Figure 1). Both are also antioxidants^[25]^ and being closely structurally related to HBA (see Figure 1) were expected to have high potential for co‐crystallisation with INCT.^[26]^

While HME is expected to be the most appropriate technique to prepare co‐crystals for industrial use, traditionally solvent evaporation has been the most commonly employed method for the preparation of pharmaceutical co‐crystals.^[27]^ Unfortunately, due to the use of organic solvents this process is ecologically unsound; it is also time consuming, with evaporation of the solvent occurring over days and weeks. Recently, thermal inkjet printing has proven to be a rapid alternative method for the preparation of pharmaceutical co‐crystals.^[28]^ Inkjet printing takes minutes to produce crystals of sufficient quantity and high enough quality for analysis. However, the materials must first be in solution before they can be printed and so the “green” problem remains. Another option is co‐grinding stoichiometric amounts of two dry powdered crystalline materials. This has been known to produce co‐crystals since as early as 1893, although it has only gained prominence in academic laboratories since 2000.^[29]^ The method has advantages over solvent evaporation and thermal inkjet printing in that it is both fast (minutes timescale) and clean/green (requires no solvent), but it produces fine powders and not the single crystals required for full structure elucidation.

In this paper, we use hyphenated DSC‐XRD to generate novel co‐crystals of INCT with HBA, HPAA and DHPAA and investigate the formation mechanisms. We further explore solvent evaporation, thermal inkjet printing, and ball milling as alternative routes to co‐crystallisation.

## Results and Discussion

### DSC‐XRD

#### INCT‐HBA

**Figure 2:**
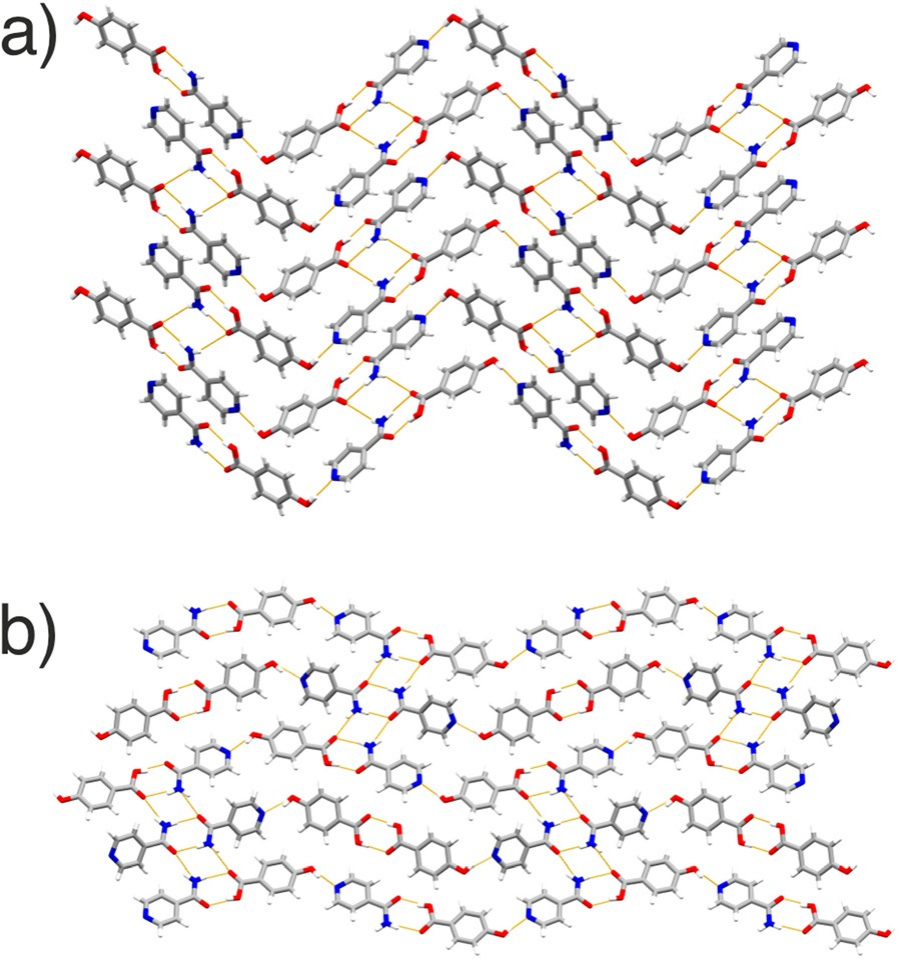
The structures of the INCT‐HBA co‐crystals, showing (a) form I and (b) form II.

Of the four systems, only INCT‐HBA has previously been shown to form co‐crystals. In this work, we were able to grow from solution both the known structure (VAKTOR, termed form I here)^[23]^ and a second, previously unreported, polymorph (form II). The key difference between form I and II of the INCT‐HBA co‐crystal is that form II is a layered hydrogen‐bonded structure, while form I contains a hydrogen‐bonded network that extends in 3D (Figure 2). A detailed discussion of the crystallography can be found in the Supporting Information (Section I.I, I.II, and I.III).

**Figure 3:**
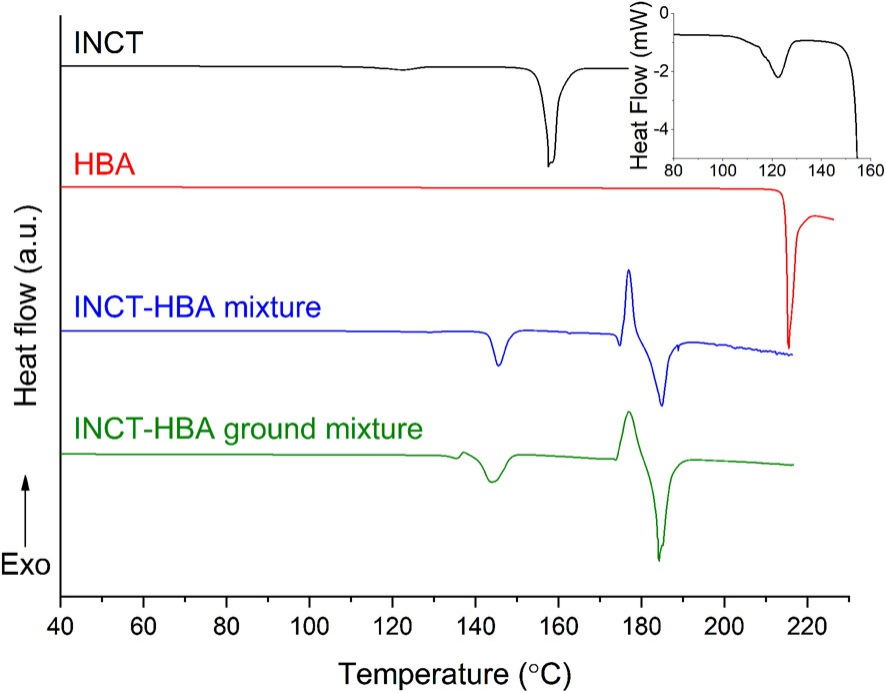
DSC thermograms recorded for INCT, HBA, a mixture of the two raw materials as received, and a mixture after they had been separately ground. Inset: an enlargement of the INCT trace in the 80–160 °C region.

DSC thermograms were recorded for two different mixtures of INCT and HBA in a 1:1 molar ratio. The first was made by weighing and mixing the two as‐supplied materials. The second was prepared by first grinding each of the two materials separately in a pestle and mortar, transferring the powders to a glass vial, and mixing for a 30 s on a vortex mixer. The traces can be compared in Figure 3. Both mixtures produced similar profiles with a number of events. The non‐ground mixture has an almost imperceptible endotherm that peaks at 129.2 °C (onset: 122.9 °C; enthalpy: 3.127 J g^−1^), followed by another larger endotherm (onset: 143.2 °C, enthalpy: 50.72 J g^−1^) peaking at 145.6 °C. The ground mixture has a similar set of events with a small endotherm at 132.6 °C (peak: 135.6 °C), immediately followed by a small overlapping exotherm (peak: ca. 137 °C), which itself is followed by an overlapping endotherm (peak: 144.0 °C). Due to the overlapping of these events accurate onset temperatures and enthalpies cannot be calculated. However, estimates show the enthalpies of the two endotherms to be 4.8 J g^−1^ and 63.7 J g^−1^ respectively.

Both mixtures then go on to experience an *endo‐*‐*exo*–*endo* event beginning at 173.9 °C (non‐ground) and 173.4 °C (ground). Again, the onsets and enthalpies of the succeeding exotherm and endotherm in both datasets cannot be accurately analysed due to the overlap of the events. However, a visual comparison indicates the enthalpies are similar and the peaks of the two events occur at 177.0 °C and 185.0 °C for the mixture and at 176.9 °C and 184.3 °C for the ground mixture. The similarity between all of the events observed must be a result of the same phase transitions occurring in each of the two mixtures.

The events observed in the data for the mixtures all occur at temperatures at which there are no events observed in the thermograms for the raw materials (Figure 3). The higher temperature events in both DSC traces must be caused by the co‐crystallisation of the materials followed immediately by their melting. It appears that the initial co‐crystallisation begins with a melt, characterised by the small endotherm immediately prior to the exotherm. It should be noted that some effects of decomposition are observed immediately following the endotherm at 145 °C. However, TGA data recorded for samples of the same two mixtures (Figure SII.1) show that, although decomposition definitely begins prior to the second set of events, at 193 °C 92.5 % of the material remains. The data strongly suggest the formation and subsequent melting of co‐crystals, despite this decomposition.

**Figure 4:**
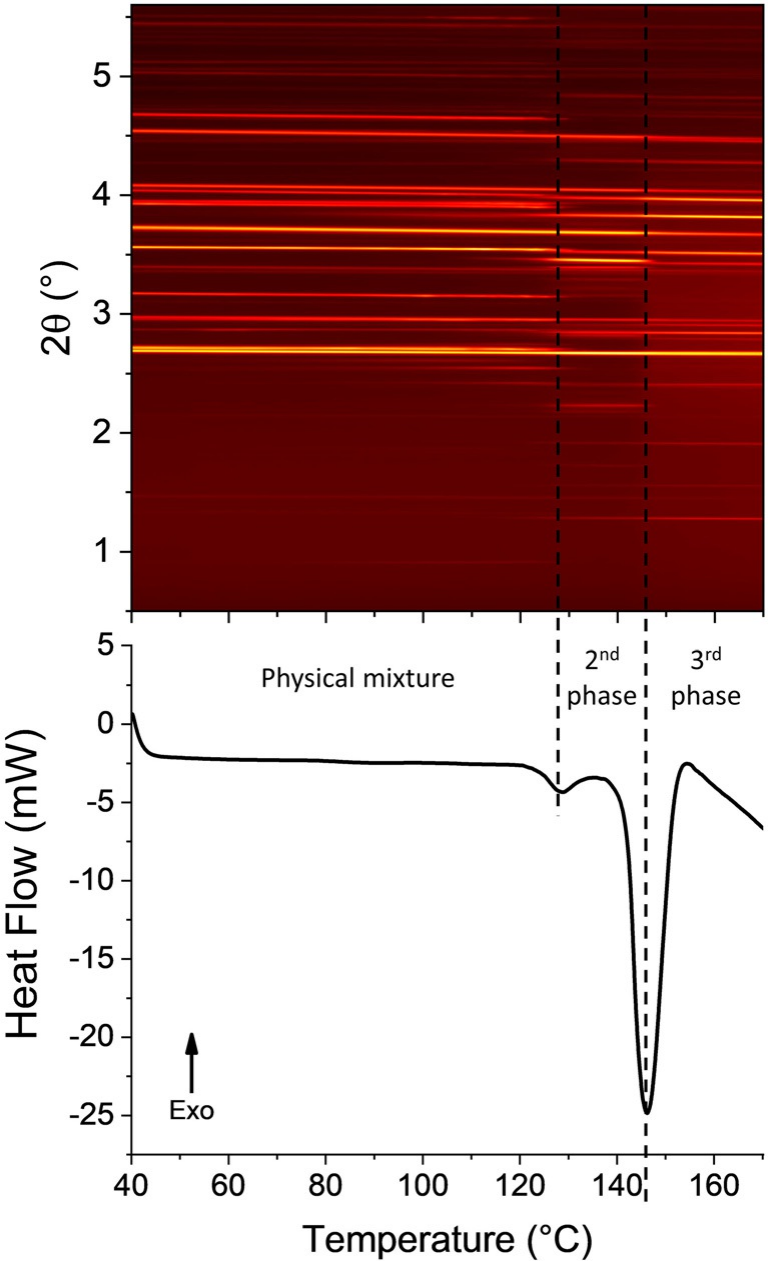
Simultaneous DSC‐XRD data for an equimolar physical mixture of INCT and HBA.

The explanation for the lower temperature group of events is less clear. Data for the raw materials do not display such an event. To understand this in more detail, a sample of the physical mixture of the two materials as received was subjected to combined DSC‐XRD analysis. The experiment was stopped at 170 °C to prevent decomposition of the sample in the DSC cell. The diffraction data and DSC thermogram (Figure 4) display two very obvious phase changes beginning at 123.4 °C (3.72 J g^−1^) and 141.9 °C (59.02 J g^−1^). Both transformations involve structural changes (resulting in distinct changes in the positions of Bragg reflections in XRD), and are endothermic. The events observed in DSC‐XRD are clearly the same as those observed by DSC, since they occur at similar temperatures and have similar associated enthalpies.

Rietveld refinement against the pattern for the physical mixture recorded at 40 °C in the DSC can be seen in Figure SII.2. Starting models used for all refinements on this system are presented in Table SII.1. Refined unit cell data can be seen in Table SII.2. The initial sample was a physical mixture of INCT form I and HBA and so the pattern recorded was expected to be a combination of the two and initial refinements were carried out using these materials. However, there was a small unidentified reflection at 2.61° and so other possible structures were introduced, including all known polymorphs of INCT, and the form I^[23]^ and II co‐crystals. The best fit was achieved when refining the structure for the form II INCT‐HBA co‐crystal. It seems that simply mixing the two materials resulted in a small amount of co‐crystallisation.

At 97 °C characteristic reflections of the form II co‐crystal are much more intense, and reflections from a fourth structure are present. The results of Rietveld refinements carried out on the pattern recorded at this temperature can be seen in Figure SII.3 and Table SII.3. The new entity is the form I co‐crystal reported by Vishweshwar.^[23]^ At 122 °C the mixture has become extremely complex and refinement (Figure SII.4; Table SII.4) reveals the presence of five species: INCT forms I and II, HBA, and both co‐crystals. Thus, rather than a simple conversion from the two initial materials to the known co‐crystal there appear to be numerous transformations occurring. INCT I and II are enantiotropically related with the I→II conversion reported by Li et al.^[30]^ to occur at 131.7 °C at a heating rate of 70 °C min^−1^. At lower heating rates this can be expected to occur at a lower temperature and samples from the same study by Li were shown to convert to form II at ca. 120 °C. Thus the presence of form II at 122 °C is not unexpected. At this temperature both forms of the co‐crystal are still present. From this pattern alone it is not possible to deduce whether the formation of form I INCT‐HBA results from the polymorphic conversion of form II or from the combination of HBA with either one or both of the forms of INCT crystals.

At 139 °C, the midpoint of the second clear phase in the contour plot and between the two endotherms in the DSC trace, reflections of INCT I and the form II co‐crystal have completely disappeared (Figure SII.5; Table SII.5). Only INCT II, HBA and form I INCT‐HBA remain and they are present in significant quantities. It should be noted that the two single component crystal species are present in a 1:1 ratio. Refinement against the final pattern recorded at 170 °C (Figure SII.6, Table SII.6) suggests that there is a small amount INCT II left in the sample. However, detailed inspection of the plots indicates that the value of 4.8 % attributed to INCT II is probably incorrect and that there is in fact no pure INCT remaining in the sample. The largest reflection in the calculated pattern for this species (3.4°) is probably a result of the software compensating for the disproportionately high intensity reflection present at the same angle in the pattern of VAKTOR. The vast majority of the remaining crystalline material can be attributed to the structure of the form I co‐crystal. However, there remains some residual HBA that has not co‐crystallised. It seems odd that there is pure HBA left but no INCT as the initial mixture was prepared at a 1:1 molar ratio and the unit cell of the co‐crystal formed contains one molecule of each. It could perhaps be the case that the initial mixture contained slightly more HBA than INCT, or the two were not perfectly homogeneously mixed in the DSC pan.

**Figure 5:**
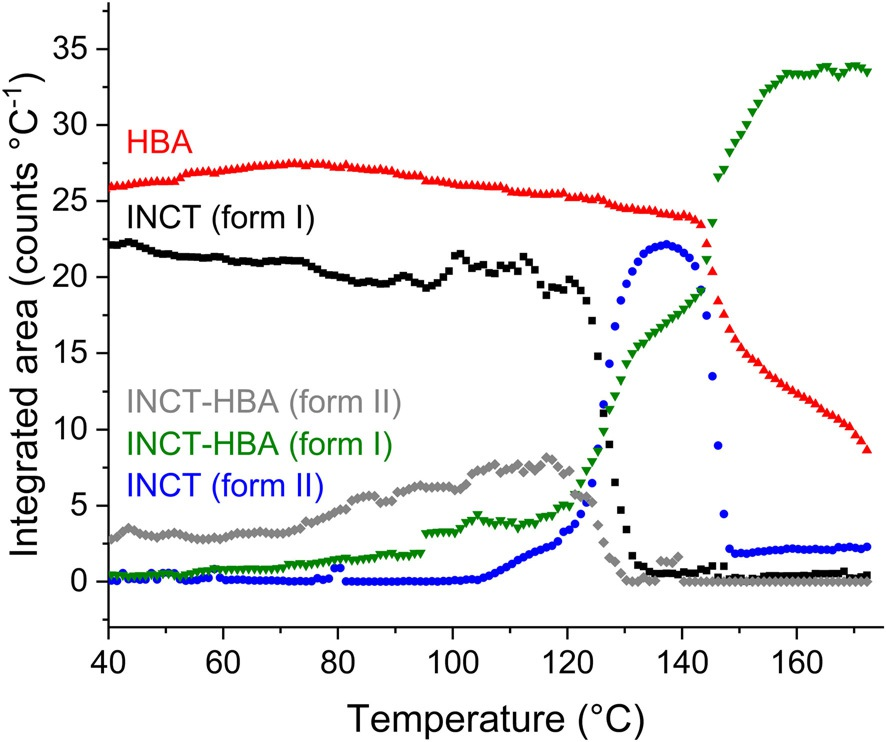
Plot of relative quantity as a function of temperature for each of the phases present in a mixture of INCT and HBA.

Plotting the total integrated intensity of each phase present as a function of time (Figure 5) it can be seen that there is little change in the overall content of HBA throughout the experiment until ca. 145 °C. There is some initial growth, presumably due to heat expansion causing more material to be lifted into the beam, followed by a gradual decline as the two co‐crystals grow. INCT I experiences a concomitant decline. At ca. 120 °C there is a sharp drop in the content of INCT I and form II INCT‐HBA, accompanied by equally sharp increases in the content of INCT II and form I INCT‐HBA. It seems that residual INCT I is converting to INCT II and form II of the co‐crystal is converting to form I. At 132 °C INCT I and form II INCT‐HBA have disappeared and the growth of form I slows before, soon after, INCT II and HBA undergo conversion to form I INCT‐HBA. However, the INCT disappears at a much higher rate than HBA.

#### INCT‐HPAA

**Figure 6:**
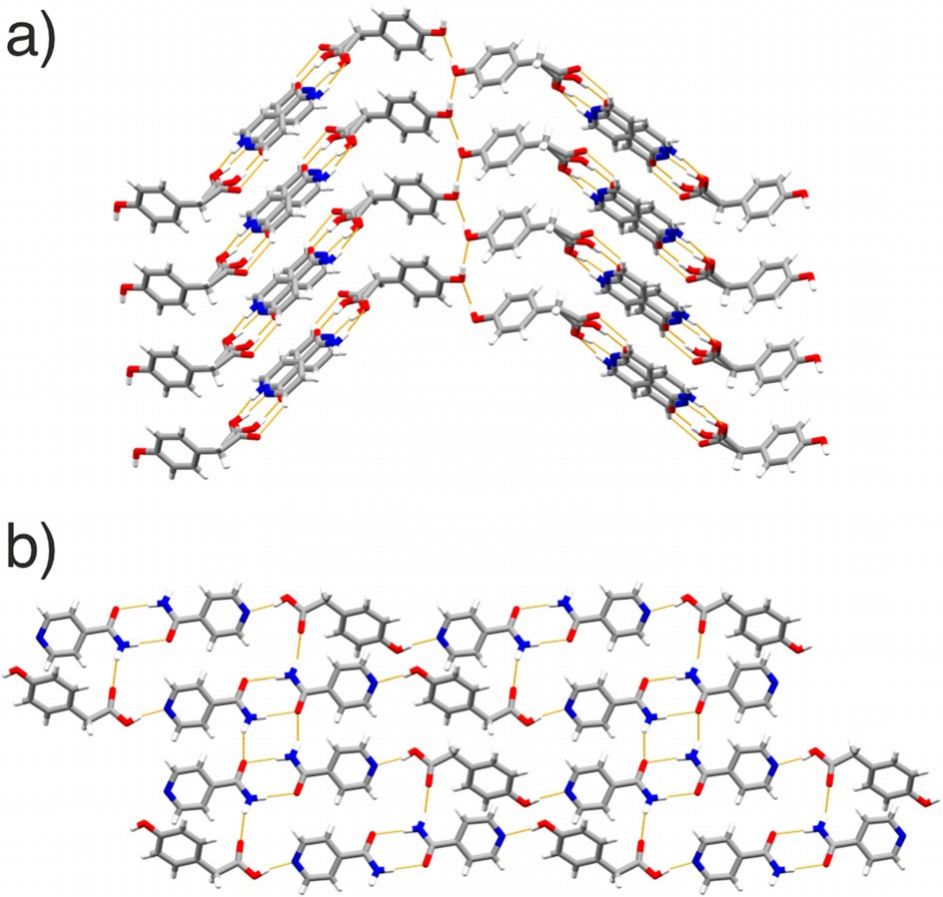
The structures of the (a) 2:1 and (b) 1:2 INCT‐HPAA co‐crystals.

Two co‐crystals with different stoichiometry (1:2 and 2:1 INCT:HPAA) were identified in this system via solvent evaporation. The 2:1 material is comprised of hydrogen‐bonded tapes, while the 1:2 INCT‐HPAA co‐crystal is based on 2D corrugated layers (Figure 6 and Section I.IV and I.V, Supporting Information).

A physical mixture of INCT and HPAA at a molar ratio of 2:1 was explored by DSC‐XRD. Initial DSC analysis can be seen in Figure SII.7. The thermogram recorded for the mixture has three endothermic events, all of which are absent from the thermograms of the two raw materials. The first is very broad and has an onset at 94.4 °C and peaks at 97.9 °C. The enthalpy associated with this event is ca. 54.9 J g^−1^ but there is a visible difference in the baseline before and after the peak due to the extended lead in to the following endotherm, and thus accurate measurement is impossible. The following events are overlapping, but the onset of the first occurs at ca. 123.6 °C, with a peak at 125.2 °C, and the second has an onset of ca. 126.3 °C and peaks at 127.7 °C. The associated enthalpies cannot be assessed. The broad nature of the first endotherm may conceal a number of events, whereas the second and third are much sharper and may indicate melting. This cannot be attributed to the melting of either of the raw materials in their most stable forms, as the temperature is too low. TGA of the mixture and the raw materials (data not shown) shows that mixing the two has a stabilising effect, with the raw materials experiencing 10 % mass loss at 193 °C (INCT) and 195 °C (HPAA), and the mixture at 205 °C. This suggests that intermolecular interactions between the components may exist.

**Figure 7:**
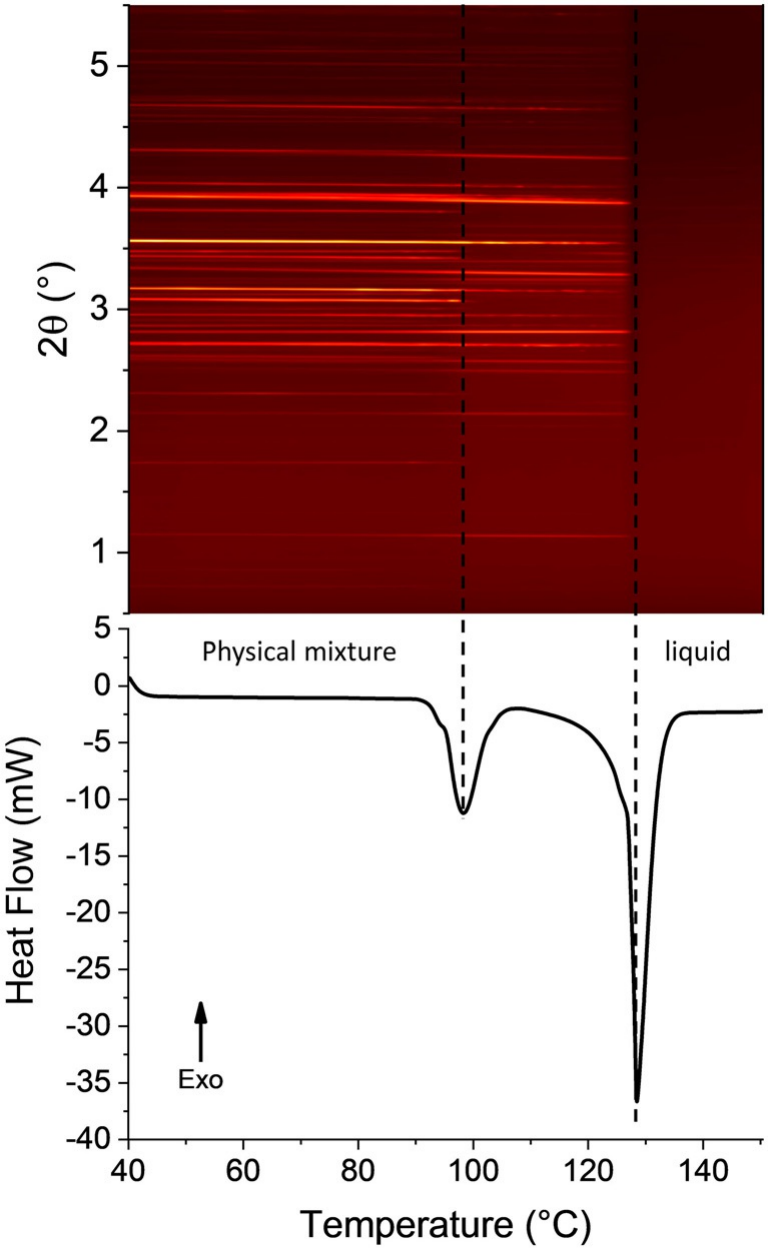
Simultaneous DSC‐XRD data for a 2:1 physical mixture of INCT and HPAA.

The same physical mixture was subjected to combined DSC‐XRD (Figure 7). The diffraction data show two major phase transitions, occurring at ca. 95 °C and ca. 125 °C. Each of these has a corresponding endotherm in the DSC trace with onsets at 94.6 °C and 127.0 °C respectively. The thermogram has a very similar form to those discussed above. The first endotherm is smaller than the second but has a small shoulder on either side, indicating that the event occurring at this temperature either occurs in multiple stages or is in fact multiple events. The second, larger, endotherm also has a small event occurring just before it. Again, this suggests a two stage process or multiple events. The total loss of Bragg reflections following the endotherm at 127.0 °C confirms the event as a melt.

Results of Rietveld refinement against the initial pattern recorded for the mixture can be seen in Figure SII.8 and Table SII.7. The calculated patterns show the initial sample to be made up of mostly INCT I and HPAA as expected, but surprisingly there was also another species present. Structures of each of the known polymorphs of INCT were included in the refinement but none improved the fit. There are no other known polymorphs of HPAA, but inclusion of the 2:1 co‐crystal showed it to be a close match to the unknown species. The fit of the calculated pattern to the data is not as good as for previous systems, with a *R*_wp_ of 0.1431. This can be partially explained by the significant difference in intensity between the data and the refinements of the reflections at 2.71° and 3.17°, characteristic of INCT. This can be attributed to the presence of large grains of material, which resulted in effects similar to preferred orientation. There are also three low intensity observed reflections at 0.73°, 2.62°, and 2.92° that are absent from the calculated pattern. The cause of these reflections is unclear. All three reflections are present from the first recorded pattern and fade from the data at the same temperature as the HPAA reflections, suggesting they are related. In contrast, the INCT reflections fade at a higher temperature and the co‐crystal reflections are present throughout the experiment. The three unexplained reflections may be the result of some impurity in the sample.

Refinement against the pattern recorded at 113 °C (Figure SII.9), between the two endotherms, has shown the sample to consist of just INCT and the co‐crystal following the first phase transition, with the majority of the material being the co‐crystal. The overall *R*_wp_ and the standard uncertainties (Table SII.8) for the co‐crystal are much lower than those calculated for the same species in the 40 °C pattern. The major reflections in the co‐crystal pattern occur at similar angles to those in the lower temperature pattern, and the three unidentified reflections are no longer present. This strengthens the argument that the co‐crystal was present in the 40 °C pattern. It appears that, like the INCT‐HBA mixture, just mixing INCT and HPAA caused some co‐crystallisation to occur.

The original stock mixture was made up at an INCT:HPAA molar ratio of 2:1, so it seems odd that at 113 °C, following the exhaustion of the HPAA, there is still a significant amount of INCT left in the sample. The integrated area under the curve for the pattern of each of the reactants at 40 °C was used to calculate the relative amounts of each material in the beam, and it seems there was an excess of INCT of around 22 % of the total sample in the beam. The remaining 12 % can be accounted for by the melting of some of the HPAA. This is visible in the diffraction patterns above ca. 100 °C as an increase in the background intensity (Figure 7), characteristic of a liquid or amorphous material.

**Figure 8:**
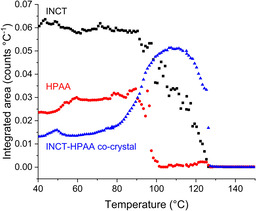
Plot of relative quantity as a function of temperature for each of the phases present in a mixture of INCT and HPAA.

The evolution of the systems present in the sample can be visualised in Figure 8. Initially the material consisted of mostly INCT, with less HPAA and a little of the co‐crystal. The content of all three remained relatively stable until around 80–90 °C when both INCT and HPAA began to decrease rapidly, whilst the co‐crystal content increases. This is the result of co‐crystallisation and coincides with the first endotherm in the DSC trace. This endotherm arises from multiple events, with both co‐crystallisation and melting occurring simultaneously. As there are three peaks it is probable that both INCT and HPAA undergo a local melt, and then recrystallise as the 2:1 co‐crystal. The combination of the two endothermic events presumably masks the exothermic crystallisation peak. Above ca. 100 °C the HPAA has been exhausted and the remaining INCT continues to melt while co‐crystal formation slows and eventually stops. Finally, the co‐crystal melts. The melting of INCT and the co‐crystal coincide with the second endotherm in the DSC trace, and the occurrence of these two melts explains the presence of the small shoulder on this event. The presence of the co‐crystal appears to destabilise INCT so that it melts at a much lower temperature than the pure crystalline powder.

#### INCT‐DHPAA

**Figure 9:**
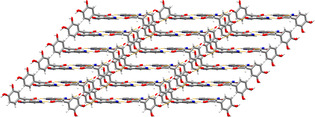
The structure of the INCT‐DHPAA co‐crystal.

The INCT‐DHPAA co‐crystal which forms from solvent evaporation is made up of staggered chains (Figure 9) with alternate INCT and DHPAA molecules linked by phenol⋅⋅⋅acid and phenol⋅⋅⋅pyridine hydrogen bonds (see also the Supporting Information, Section I.VI).

**Figure 10:**
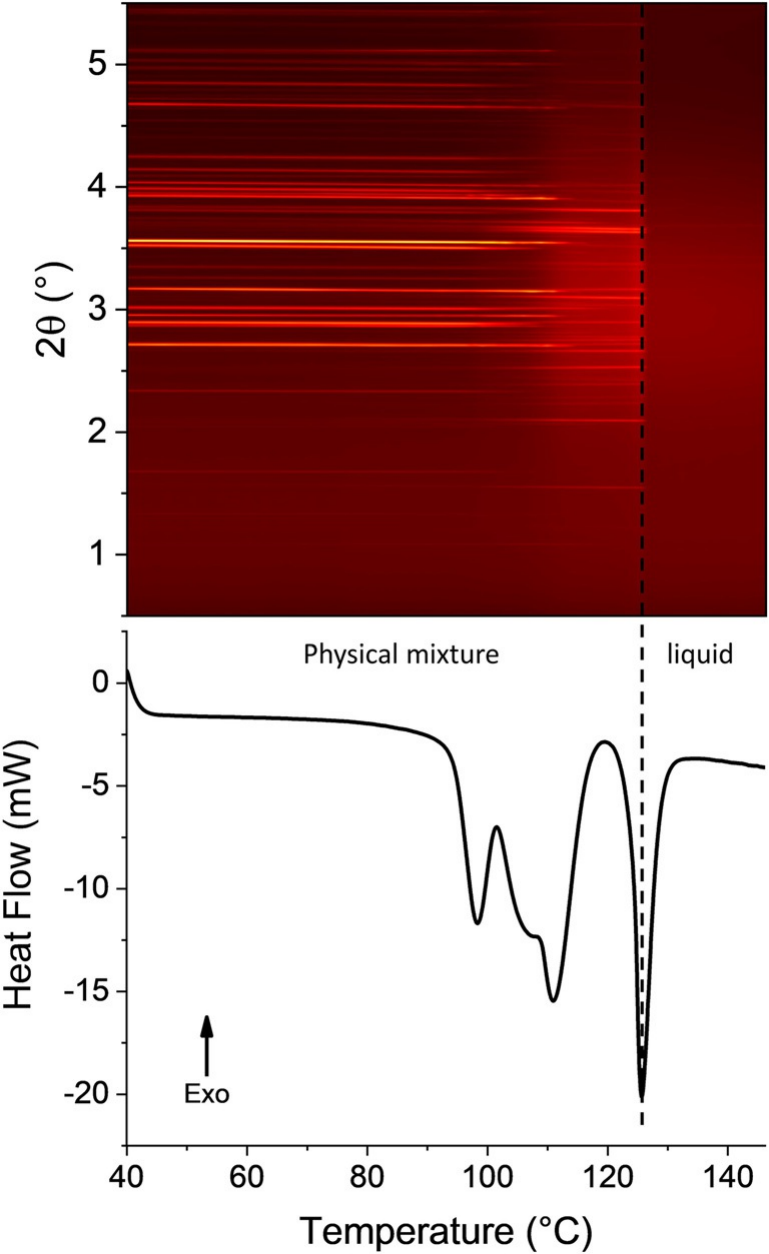
Simultaneous DSC‐XRD data for a 1:1 physical mixture of INCT and DHPAA.

Diffraction patterns collected during simultaneous DSC‐XRD experiments on a binary mixture of INCT and DHPAA (Figure 10) appear to show the occurrence of two phase transitions. Reflections of the phases present before and after the first transition overlap in a broad range of temperatures (ca. 95–115 °C). From these data alone it would appear that a single event is occurring. However, the DSC thermogram shows that there are in fact at least three endothermic events taking place between 90 °C and 120 °C. The final transition is much clearer and is represented by the complete loss of Bragg reflections and a sharp endotherm, and is the final melting of the sample.

Closer examination of the diffraction data between 80 °C and 120 °C does not offer any explanation of the multiple events in the thermogram. The Bragg reflections present at the beginning of the experiment are continuous until their disappearance at ca. 120 °C and the same is true of the reflections representing the second crystalline phase from their appearance at ca. 90 °C until their disappearance at 126 °C. This then suggests that the multiple endotherms are probably caused by different stages of the same process as the crystal structures of the two materials are first disrupted before realigning into the structure of the co‐crystal. Figure SII.10 and Table SII.9 show refinement data for the initial pattern recorded for the sample at 40 °C. Using the structures of the two raw materials achieved a very good fit with an overall R_wp_ of 0.0556.

**Figure 11:**
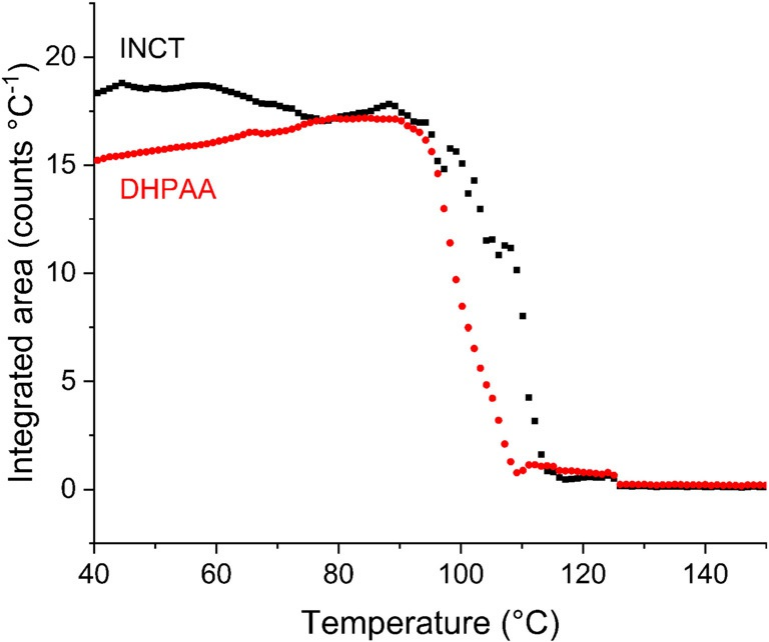
Plot of relative quantity as a function of temperature for each of the phases present in a mixture of INCT and DHPAA.

The pattern recorded at 122 °C (Figure SII.11), after the first phase transition, could not be fitted by the structures of any of the known polymorphs of the two raw materials or that of the INCT‐DHPAA co‐crystal grown by solvent evaporation. Many of the reflections in the pattern collected by DSC‐XRD occur at similar angles to reflections in the predicted pattern (Figure SII.11), but there are significant absences (in particular the two main reflections at 3.02° and 3.76°). The new structure must be either the result of co‐crystal formation or the crystallisation of a new polymorph of one of the two raw materials. There is also a high background that emerges at the same time as the third endotherm in DSC (Figure 11). This is not present in the lower temperature patterns, and indicates the presence of some amorphous or melted material.

Plotting the integrated area under the calculated pattern of each species as a function of temperature (Figure 11) it can be seen that the content of both of the raw materials remains relatively constant until ca. 95 °C, at which point both begin to decline. This coincides with the onset of the first endotherm in the thermogram (94.2 °C). However, the total disappearance of DHPAA and INCT does not occur until 108.8 °C and 113.9 °C respectively. These endpoints match closely the minima of the second (ca. 108 °C) and third (111.05 °C) endotherms. The overlapping nature of these events and the likelihood of an invisible exothermic event relating to crystallisation means that a confident assignment is not possible. That said, it can be deduced that the disappearance of both structures from the sample is not due simply to melting, as the raw materials have melting points at temperatures in excess of 120 °C. It is, therefore, likely to be the result of a conversion from one solid form to another. It cannot be ascertained from these data whether that form is a co‐crystal or a new polymorph of one of the two individual components.

### Inkjet printing

#### INCT‐HBA

**Figure 12:**
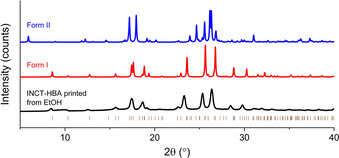
Diffraction pattern recorded for printed crystals of INCT‐HBA, and calculated patterns for the form I co‐crystal (VAKTOR) and the form II system grown from solvent evaporation. Tick marks show the positions of allowed reflections for form I.

The diffraction patterns recorded for crystals obtained from printing have some significant differences from those of the raw materials (Figure SII.12). When compared to the predicted powder pattern of the structure of the form I co‐crystal (VAKTOR)^[23]^ and that of form II it is clear that the pattern of the printed crystals corresponds to the form I system (Figure 12). The data recorded here are of relatively low resolution due to the small crystallite and sample size, but all of the reflections occur at the expected 2θ angles and the intensity ratios are very similar to those of form I. The slight discrepancy between the calculated pattern for form I and that recorded for the printed sample is attributed to the two datasets being recorded at −196 °C and room temperature, respectively. The formation of this co‐crystal is confirmed DSC, TGA, and IR spectroscopy (Figure SII.13).

#### INCT‐HPAA

Crystals could be printed from solutions of INCT and HPAA (molar ratio: 2:1) in either ethanol or ethanol and water (Figure SII.14). All the solutions explored yielded crystals with the same structure. The patterns obtained clearly do not match those of the individual as‐supplied co‐formers (Figure SII.14), nor of any known polymorphs of the co‐formers (data not shown). The printed crystals‘ pattern agrees well with the predicted pattern of the 2:1 INCT‐HPAA co‐crystal structure (Figure SII.15). Bragg reflections for the printed crystals occur at slightly lower angles than for the single crystal data, but this is simply a result of the temperature difference between the measurements. The formation of the co‐crystal was verified by DSC, TGA, and IR spectroscopy (Figure SII.16).

#### INCT‐DHPAA

Equimolar solutions of INCT and DHPAA in either ethanol or ethanol/water mixtures all produced crystals with the same structure (Figure SII.17). A comparison of patterns recorded for the printed crystals, the raw materials separately, and a physical mixture of the two can be seen in Figure SII.18. The pattern for the printed crystals is clearly very different to those of the raw materials, but matches closely with the INCT‐DHPAA co‐crystal described above (Figure SII.19). Successful formation of a co‐crystal was confirmed by DSC, TGA, and IR spectroscopy (Figure SII.20).

### Milling

#### INCT‐HBA

**Figure 13:**
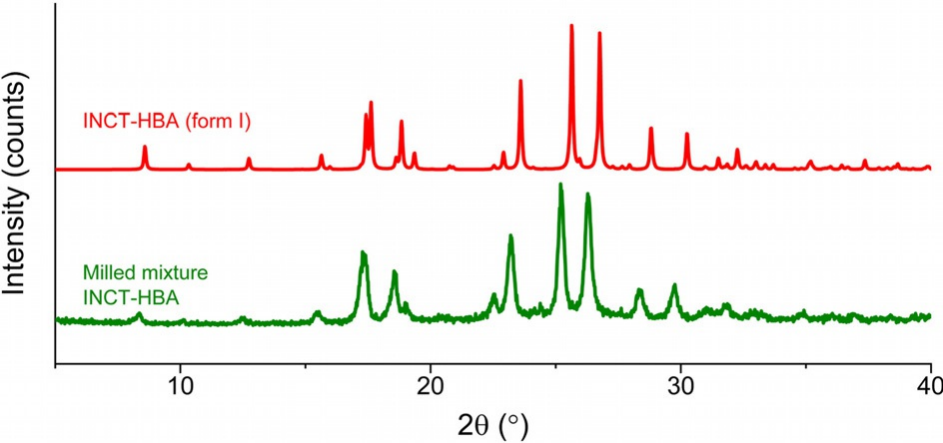
Diffraction pattern recorded for the product of ball milling INCT and HBA and the simulated pattern of INCT‐HBA form I.

The powder produced by ball milling an equimolar mixture of INCT and HBA at 20 Hz for 15 min was analysed by DSC and XRD. The DSC data (Figure SII.21) show one clear endothermic event with an onset at 183.3 °C, after which the material begins to degrade. This endotherm occurs at the same temperature as observed for the printed crystals (181.6 °C), and so again it appears that a co‐crystal has formed. XRD analysis (Figure 13) resulted in a pattern very similar to that of the previously reported form I co‐crystal,^[23]^ albeit with an overall shift in reflection positions to lower angles. The intensity ratios are very similar, as are the relative peak positions. The similarity of the two patterns suggests that the structures of the two samples are the same. The shift in angles of diffraction can be attributed to the difference in temperature of the two samples. The FTIR spectrum recorded for the milled sample (Figure SII.22) is almost identical to the spectrum recorded for the printed crystals, which again supports the conclusion that a co‐crystal has formed upon milling.

#### INCT‐HPAA

The results obtained after milling mixtures of INCT and HPAA in a 2:1 molar ratio are consistent with the production of a co‐crystal. The DSC data (Figure SII.23) display a single endotherm with onset at 126.3 °C, the same temperature as the printed crystals (125.9 °C). The XRD pattern of the product of milling matches closely with that predicted for the 2:1 co‐crystal (see Figure SII.24). The FTIR spectra (Figure SII.25) are also very similar for the milled and printed samples, thus confirming that the same co‐crystal has been generated in both cases.

#### INCT‐DHPAA

Contrary to the previous examples, the data for the milled mixture of INCT and DHPAA are not identical to those from printing. There are distinct differences noted in the melting points in DSC (Figure SII.26) and the reflection positions and intensities in XRD (Figure SII.27). The latter do not suggest a simple physical mixture of the two components, but rather that a second co‐crystal system has been generated. A comparison of the XRD data from the milled system and the phase observed at 122 °C in the DSC‐XRD experiment suggest that the two materials are the same, however (Figure SII.28). The full details of this are not yet known, and to date we have been unable to produce single crystals of this material. Investigations are ongoing.

## Discussion

This study reports a systematic exploration of the formation of co‐crystals containing INCT and a series of APIs. It is clear that the formation of co‐crystals is a complicated and multi‐faceted process. In the case of INCT‐HBA, two polymorphic co‐crystals can be obtained from solvent evaporation experiments. Both form I and form II are observed during thermal synthesis, with form II forming first and converting to form I upon continued heating. Ink jet printing and ball milling yielded only form I of the INCT‐HBA co‐crystal. In the case of INCT‐HPAA, co‐crystals with two different stoichiometries (2:1 and 1:2) formed after solvent evaporation. Inkjet printing and ball milling the two co‐formers at a 2:1 molar ratio resulted in the 2:1 co‐crystal, as did a thermal treatment. A 1:1 INCT‐DHPAA co‐crystal forms upon solvent evaporation and from ink jet printing, but milling and thermal synthesis result in a different material, the structure of which could not be determined.

What is stark from these results is that different routes of co‐crystallisation yield different products, and that this is highly system‐specific. While it is possible to generate co‐crystals via ink jet printing and milling approaches, and hence it can be concluded that these routes do have the potential to be used for pharmaceutical manufacturing, the complex polymorphism which arises in co‐crystallisation is a complicating factor, and great care will be required to ensure that the desired material is generated. Further, when developing medical products in the form of co‐crystals, it will be necessary for pharmaceutical companies to employ a range of synthetic processes during preformulation studies in order to ensure that the polymorphic landscape is well understood. Crucially, the standard solvent screening method alone does not allow identification of all the phases possible for a co‐former system.

DSC‐XRD experiments reveal that a range of simultaneous processes occur during the thermal synthesis of co‐crystals. Rather than simple solid‐solid or melt‐crystallisation transformations, we observe the different co‐formers to undergo polymorphic transitions themselves, and to melt at different points. It is also notable that simple mixing appears sufficient to cause a small amount of co‐crystal formation. These findings will be important to pharmaceutical manufacturers, given that the most likely route to generate co‐crystals in industry is HME, a thermally‐mediated fabrication method.

## Conclusion

Four new co‐crystals containing isonicotinamide (INCT) with 4‐hydroxybenzoic acid (HBA), 4‐hydroxyphenylacetic acid (HPAA) and 3,4‐dihydroxyphenylactic acid (DHPAA) were synthesised using a solvent evaporation approach in this work. These include a new polymorph of the 1:1 INCT‐HBA co‐crystal, 2:1 and 1:2 INCT‐HPAA materials, and a 1:1 INCT‐DHPAA system. Simultaneous DSC‐XRD experiments reveal that the formation of co‐crystals during heating is an extremely complex process and, in addition to co‐crystallisation, conversions between polymorphs of the co‐formers were also observed. Thermal methods, inkjet printing, solvent evaporation and milling all produced co‐crystals, but there were differences in the phases obtained from the different methods. In the case of INCT‐HBA, both co‐crystal polymorphs were observed during heating, but only the previously reported form I resulted from inkjet printing and milling. The 2:1 co‐crystal of INCT‐HPAA was observed from all synthetic routes, but with the INCT‐DHPAA system while the same co‐crystal was seen from solvent‐based approaches (evaporation and printing) a different material was obtained thermally and from milling. Overall, it is clear that a wide range of approaches need to be implemented in the development of pharmaceutical co‐crystals to ensure that the polymorphic landscape is fully understood.

## Experimental Section

Full details of the experimental procedures used in this work are given in the Supporting Information (Section III).

## Conflict of interest

The authors declare no conflict of interest.

## Supporting information

As a service to our authors and readers, this journal provides supporting information supplied by the authors. Such materials are peer reviewed and may be re‐organized for online delivery, but are not copy‐edited or typeset. Technical support issues arising from supporting information (other than missing files) should be addressed to the authors.

Supplementary

## References

[1] N. Shan , M. J. Zaworotko , Drug Discovery Today 2008, 13, 440–446. 18468562 10.1016/j.drudis.2008.03.004

[2] M. Rodrigues , J. Lopes , M. Sarraguça , Molecules 2018, 23, 3263. 10.3390/molecules23123263 PMC6321374 30544751

[3] S. Kumar , A. Nanda , Mol. Cryst. Liq. Cryst. 2018, 667, 54–77.

[4] M. Malamatari , S. A. Ross , D. Douroumis , S. P. Velaga , Adv. Drug Delivery Rev. 2017, 117, 162–177. 10.1016/j.addr.2017.08.006 28811184

[5] A. Clout , A. B. M. Buanz , T. J. Prior , C. Reinhard , Y. Wu , D. O′Hare , G. R. Williams , S. Gaisford , Anal. Chem. 2016, 88, 10111–10117. 27642771 10.1021/acs.analchem.6b02549

[6] A. E. Clout , A. B. M. Buanz , S. Gaisford , G. R. Williams , Chem. Eur. J. 2018, 24, 13573–13581. 29979477 10.1002/chem.201802368 PMC6175174

[7] R. Telford , C. C. Seaton , A. Clout , A. Buanz , S. Gaisford , G. R. Williams , T. J. Prior , C. H. Okoye , T. Munshi , I. J. Scowen , Chem. Commun. 2016, 52, 12028–12031. 10.1039/c6cc05006a 27510730

[8] S. Askin , J. K. Cockcroft , L. S. Price , A. D. Gonçalves , M. Zhao , D. A. Tocher , G. R. Williams , S. Gaisford , D. Q. M. Craig , Cryst. Growth Des. 2019, 19, 2751–2757.

[9] D. Douroumis , S. A. Ross , A. Nokhodchi , Adv. Drug Delivery Rev. 2017, 117, 178–195. 10.1016/j.addr.2017.07.008 28712924

[10] M. Gajda , K. P. Nartowski , J. Pluta , B. Karolewicz , Int. J. Pharm. 2019, 558, 426–440. 30664997 10.1016/j.ijpharm.2019.01.016

[11] J.-R. Wang , X. Yu , C. Zhou , Y. Lin , C. Chen , G. Pan , X. Mei , Bioorg. Med. Chem. Lett. 2015, 25, 1036–1039. 25630224 10.1016/j.bmcl.2015.01.022

[12] Y. Kang , J. Gu , X. Hu , J. Mol. Struct. 2017, 1130, 480–486.

[13] K. V. Drozd , A. N. Manin , A. V. Churakov , G. L. Perlovich , Eur. J. Pharm. Sci. 2017, 99, 228–239. 28011126 10.1016/j.ejps.2016.12.016

[14] J. Li , L. Wang , Y. Q. Ye , X. Fu , Q. Ren , H. Zhang , Z. Deng , Eur. J. Pharm. Sci. 2016, 85, 47–52. 26836368 10.1016/j.ejps.2016.01.029

[15] Q.-Z. Zeng , J. Ouyang , S. Zhang , L. Zhang , Eur. J. Pharm. Sci. 2017, 102, 140–146. 28254392 10.1016/j.ejps.2017.02.035

[16] P. Sanphui , S. S. Kumar , A. Nangia , Cryst. Growth Des. 2012, 12, 4588–4599.

[17] S. Basavoju , D. Bostrom , P. Velaga , Cryst. Growth Des. 2006, 6, 2699–2708.

[18] N. Rajesh Goud , R. A. Khan , A. Nangia , CrystEngComm 2014, 16, 5859.

[19] S. Domingos , A. Fernandes , M. T. Duarte , M. F. M. Piedade , Cryst. Growth Des. 2016, 16, 1879–1892.

[20] P. Sanphui , G. Bolla , A. Nangia , Cryst. Growth Des. 2012, 12, 2023–2036.

[21] N. B. Báthori , A. Lemmerer , G. A. Venter , S. A. Bourne , M. R. Caira , Cryst. Growth Des. 2011, 11, 75–87.

[22] C. B. Aakeröy , A. M. Beatty , B. A. Helfrich , Angew. Chem. Int. Ed. 2001, 40, 3240–3242; 10.1002/1521-3773(20010903)40:17<3240::AID-ANIE3240>3.0.CO;2-X 29712056

[23] P. Vishweshwar , A. Nangia , V. M. Lynch , CrystEngComm 2003, 5, 164–168.

[24] J.-Y. Cho , J.-H. Moon , K.-Y. Seong , K.-H. Park , Biosci. Biotechnol. Biochem. 1998, 62, 2273–2276. 9972252 10.1271/bbb.62.2273

[25] I. Biskup , I. Golonka , A. Gamian , Z. Sroka , Postepy Hig. Med. Dosw. 2013, 67, 958–963. 10.5604/17322693.1066062 24088539

[26] M. K. Corpinot , D.-K. Bučar , Cryst. Growth Des. 2019, 19, 1426–1453.

[27] S. Aitipamula , P. S. Chow , R. B. H. Tan , CrystEngComm 2009, 11, 889.

[28] A. B. M. Buanz , R. Telford , I. J. Scowen , S. Gaisford , CrystEngComm 2013, 15, 1031–1035.

[29] T. Friščić , W. Jones , Cryst. Growth Des. 2009, 9, 1621–1637.

[30] J. Li , S. A. Bourne , M. R. Caira , Chem. Commun. 2011, 47, 1530–1532. 10.1039/c0cc04117c 21088781

