# Supplementary material for: Mechanistic In Situ and Ex Situ Studies of Phase Transformations in Molecular Co‐Crystals
Source: Chemistry. 2020 Oct 7;26(64):14645–53. doi: 10.1002/chem.202002267 (PMC7756291; doi:10.1002/chem.202002267)
Supplement: Supplementary file 1 — Supplementary [file CHEM-26-14645-s001.pdf]

Chemistry–A European Journal

Supporting Information

Mechanistic In Situ and Ex Situ Studies of Phase Transformations in Molecular Co-Crystals

Alexander E. Clout,[a] Asma B. M. Buanz,[a] Yuying Pang,[a] Wing-Mei Tsui,[a] Dongpeng Yan,[b] Gary Parkinson,[a] Timothy J. Prior,[c] Dejan-Krešimir Bučar,[d] Simon Gaisford,*[a] and Gareth R. Williams*[a]

I. Crystallography

I.I Single crystal X-ray diffraction measurements

The diffraction data for the INCT-HBA (Form I), INCT-HPAA (1:2), INCT-HPAA (2:1) and the INCT-DHPAA co-crystals were collected on a four-circle Agilent SuperNova (Dual Source) single crystal X-ray diffractometer using a micro-focus CuK_{α} X-ray beam (λ = 1.54184 Å) and an Atlas CCD detector. The sample temperatures were controlled with an Oxford Instruments cryojet. All data were processed using the $CrysAlis^{Pro}$ programme package from Rigaku Oxford Diffraction.[1] The crystal structures were solved with the ShelXT programme[2] and refined by least squares on the basis of F^2 with the ShelXL programme.[3] Both programmes were used within the $Olex^2$ software suite.[4] All non-hydrogen atoms were refined anisotropically by the full-matrix least-squares method. Hydrogen atoms affiliated with oxygen and nitrogen atoms were refined isotropically [$U_{iso}(H) = 1.5U_{eq}(O/N)$] in geometrically constrained positions. Hydrogen atoms associated with carbon atoms were refined isotropically [$U_{iso}(H) = 1.2U_{eq}(C)$] in geometrically constrained positions. The atom coordinates of the disordered isonicotinamide molecule in the INCT-HPAA (1:2) co-crystal were refined using the FRAG/FEND instructions in ShelXL and the atomic coordinates of a planar isonicotinamide molecule, which were retrieved from the crystal structure of the isonicotinamide formamide solvate (CSD GAVHER, data collection temperature: 150 K).[5]

The data for INCT-HBA (Form II) were collected on an Oxford Diffraction Xcalibur diffractometer equipped with a Titan CCD detector and a CuK_{α} X-ray beam (λ = 1.54184 Å). The data were obtained at 107 K and processed using the $CrysAlis^{Pro}$ software package. The crystal structure was solved and refined using the ShelXT[6] and ShelXL[3] programs, as described above.

The crystallographic parameters for all four co-crystals are detailed in Table SI.1.

Table SI.1. Crystallographic and refinement parameters**.**

	**INCT-HBA, 1:1 Form I**	**INCT-HBA, 1:1 Form II**	**INCT-HPAA, 1:2**	**INCT-HPAA, 2:1**	**INCT-DHPAA 1 :1**
empirical formula	$(C_6H_6N_2O)\cdot(C_7H_6O_3)$	$(C_6H_6N_2O)\cdot(C_7H_6O_3)$	$(C_6H_6N_2O)\cdot(C_8H_8O_3)_2$	$(C_6H_6N_2O)_2\cdot(C_8H_8O_3)$	$(C_6H_6N_2O)\cdot(C_8H_8O_4)$
M_r / g mol^{-1}	260.25	260.25	426.41	396.40	290.27
crystal system	monoclinic	monoclinic	monoclinic	triclinic	triclinic
space group	$P2_1/n$	$P2_1/c$	$P2_1/n$	$P\overline{1}$	$P\overline{1}$
a / Å	6.06690(10)	10.5720(3)	5.0202(3)	6.7637(3)	5.4364(3)
b / Å	9.3862(2)	29.8495(9)	4.9148(2)	12.3551(4)	9.8327(5)
c / Å	20.6448(3)	7.6215(3)	41.411(2)	12.6711(5)	12.9752(8)
α / °	90	90	90	111.899(3)	72.023(5)
β / °	95.235(2)	97.887(2)	90.491(5)	96.696(3)	83.214(5)
γ / °	90	90	90	99.520(3)	89.557(4)
V / Å^3	1170.72(4)	2382.36(14)	1021.71(9)	950.37(7)	654.80(7)
Z	4	8	2	2	2
ρ_{calc} / g cm^{-3}	1.477	1.451	1.386	1.385	1.472
T / K	150.0(1)	107(2)	150.0(1)	150.0(1)	150.0(1)
μ / mm^{-1}	0.936	0.920	0.874	0.847	0.957
F(000)	544	1088	448	416	304
crystal size / mm^3	0.40 × 0.34 × 0.19	0.17 × 0.07 × 0.02	0.099×0.060×0.002	0.23 × 0.17 × 0.08	0.32 × 0.11 × 0.05
Radiation	CuK_α (λ = 1.54184)	CuK_α (λ = 1.54184)	CuK_α (λ = 1.54184)	CuK_α (λ = 1.54184)	CuK_α (λ = 1.54184)
2 range for data collection / °	4.301 − 66.551	2.961− 62.737	4.271 − 73.269	3.833 − 66.590	3.608 − 66.600
index ranges	$-7 \leq h \leq 6$<br>$-11 \leq k \leq 11$<br>$-24 \leq l \leq 24$	$-9 \leq h \leq 12$<br>$-23 \leq k \leq 33$<br>$-8 \leq l \leq 7$	$-6 \leq h \leq 5$<br>$-5 \leq k \leq 5$<br>$-51 \leq l \leq 50$	$-8 \leq h \leq 7$<br>$-14 \leq k \leq 14$<br>$-15 \leq l \leq 15$	$-6 \leq h \leq 6$<br>$-11 \leq k \leq 11$<br>$-15 \leq l \leq 14$
number of collected reflections	17057	7158	8728	13863	9096
unique reflections	2071	3665	1960	3337	2328
number of unique reflections	1884 [$I > 2\sigma(I)$]	2954 [$I > 2\sigma(I)$]	1570 [$I > 2\sigma(I)$]	2995 [$I > 2\sigma(I)$]	1924 [$I > 2\sigma(I)$]
R_{int}	0.0262	0.0231	0.0339	0.0277	0.0418
$R(F)$, $F > 2\sigma$(F)	0.0330	0.0420	0.0494	0.0350	0.0406
$wR(F^2)$, $F > 2\sigma$(F)	0.0855	0.1059	0.1180	0.0960	0.1059
$R(F)$, all data	0.0363	0.0553	0.0631	0.0388	0.0506
$wR(F^2)$, all data	0.0885	0.1185	0.1277	0.1001	0.1167
Δ_r (max., min.) e Å^{-3}	0.179/−0.189	0.212/−0.200	0.174/−0.228	0.219/−0.170	0.221/−0.233
CCDC deposition number	1992677	1923377	1992680	1992678	1992679

I.II INCT-HBA 1:1, form I

The crystal structure of the 1:1 INCT-HBA co-crystal was first reported by Vishweshwar *et al.* (CSD reference code: VAKTOR)[7] and was re-determined during this study. The material crystallises in the in the monoclinic space group $P2_1/n$ with both one molecule of INCT and HBA in the asymmetric unit. The two co-crystal components interact *via* N–H(amide)···O(carboxyl) and O–H(hydroxyl)···N(pyridyl) hydrogen bonds, which define $R_2^2(8)$ and $D(2)$ hydrogen bond patterns. INCT and HBA form one-dimensional zigzag-shaped molecular chains, wherein the INCT and HBA links assume alternating positions. Neighbouring chains, being related by inversion centres, assemble into two-dimensional molecular sheets that are held together by N–H(amide)···O(carboxyl) hydrogen bonds, which establish a $R_4^2(8)$ pattern (Figure SI.1a,b). These sheets stack to form a three-dimensional structure that is sustained by N–H(amide)···O(carboxyl) hydrogen bonds and characterised by $R_2^4(8)$ patterns (Figure SI.1c). The three-dimnesional structure is further sustained by C–H···O, C–H···π and π···π interactions.

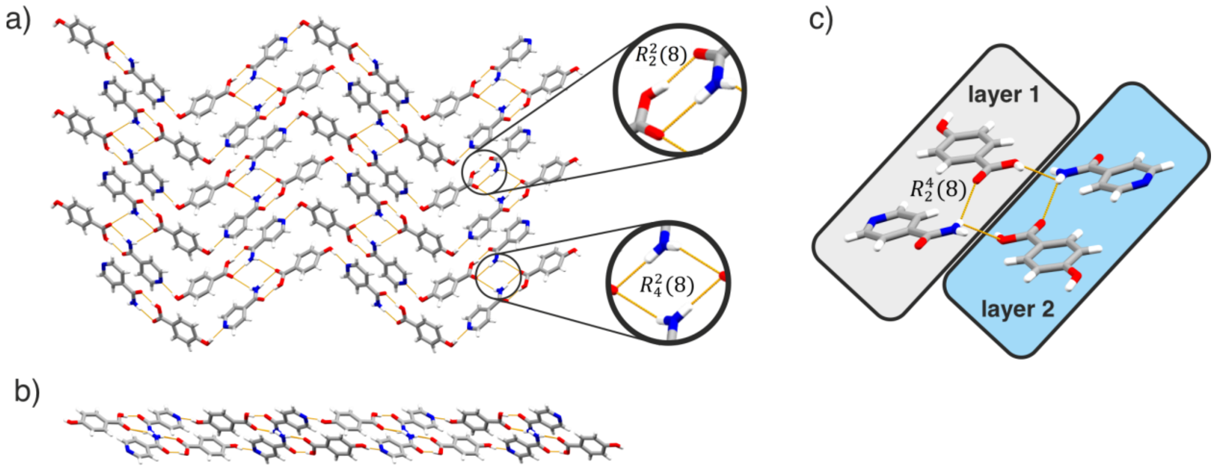

Figure SI.1. Single crystal X-ray structure of a two-dimensional molecular INCT-HBA sheet in the form I co-crystal viewed: a) along the (108) and (010) crystallographic planes and b) along the crystallographic axis *b*. A perspective view of a set of hydrogen bonds that link two stacked molecular sheets is shown in c).

I.III INCT-HBA 1:1, form II

INCT and HBA co-crystallise in the monoclinic space group $P2_1/c$ with one molecule of INCT and one molecule of HBA in the asymmetric unit. The co-crystal components form two types of zig-zag-shaped molecular chains (Figure SI.2). The first chain type is, from a supramolecular perspective, identical to those found in Form I, wherein the alternating INCT and HBA links are held together by $R_2^2(8)$ hydrogen-bond patterns, being defined by N–H(amide)···O(carboxyl) and O–H(carboxyl)···O(amide) hydrogen bonds, and by $D(2)$ patterns, being defined by O–H (hydroxyl)···N(pyridyl) hydrogen bonds. In the second chain type, the links are based on hydrogen-bonded INCT dimers and HBA dimers, rather than on individual INCT and HBA molecules. The INCT dimers are held together by N–H(amide)···O(amide) interactions and form $R_2^2(8)$ patterns, while the HBA dimers are sustained by O–H(carboxyl)···O(carboxyl) hydrogen bonds that define another $R_2^2(8)$ pattern (Figure SI.2a).

The INCT and HBA dimers are linked in an alternating fashion through O–H(hydroxyl)···N(pyridyl) hydrogen bonds, which establish a $D(2)$ pattern. Chains of both types form two-dimensional hydrogen bonded sheets that are sustained by N–H(amide)···O(amide) and N–H(amide)···O(carboxyl) hydrogen bonds (describing a $R_4^2(8)$ pattern, Figure SI.2a) and a range of weaker C–H···O and C–H···π interactions. The sheets (Figure SI.2b) are stacked into a three-dimensional structure that is stabilised by C–H···π and π···π interactions.

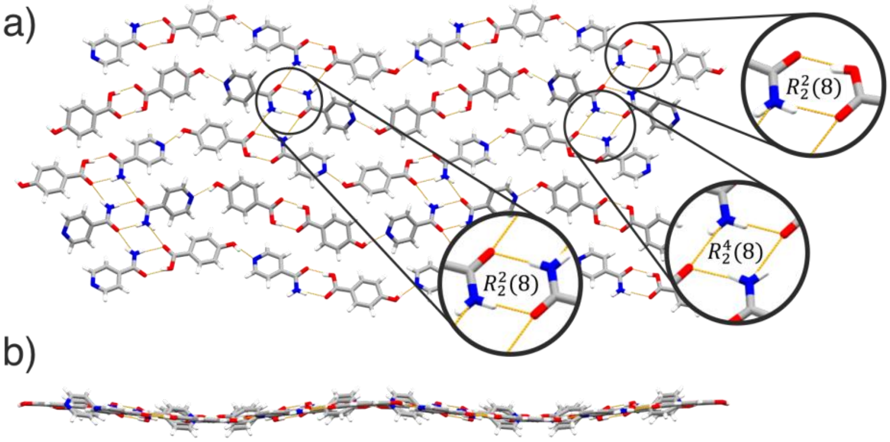

Figure SI.2. Single crystal X-ray structure of a two-dimensional molecular INCT-HBA sheet in the form II co-crystal viewed: a) along the crystallographic axis *c*, and b) along the crystallographic planes (102) and (010).

I.IV INCT-HPPA 1:2

INCT and HPAA co-crystallise in the monoclinic space group $P2_1/n$ with one molecule of INCT and two molecules of HPAA in the asymmetric unit. Each INCT molecule (being disordered around an inversion centre) interacts with two HPAA molecules; with one through N–H(amide)···O(carboxyl) and O–H(carboxyl)···O(amide) hydrogen bonds (thus constituting an $R_2^2(8)$ pattern) and another one via O–H(carboxyl)···N(pyridyl) and C–H(pyridyl)···O(carboxyl) interactions (giving an $R_2^2(7)$ pattern). The three-component INCT:HPAA aggregates further assemble into a two-dimensional corrugated hydrogen-bonded sheet *via* O–H(hydroxyl)···O(hydroxyl) hydrogen bonds that form a $C(2)$ pattern (Figure SI.3). The two-dimensional sheets are stacked and interact with each other via $D(2)$ N–H(amide)···O(carboxyl) hydrogen bonds, as well as weaker C–H···O and π···π interactions.

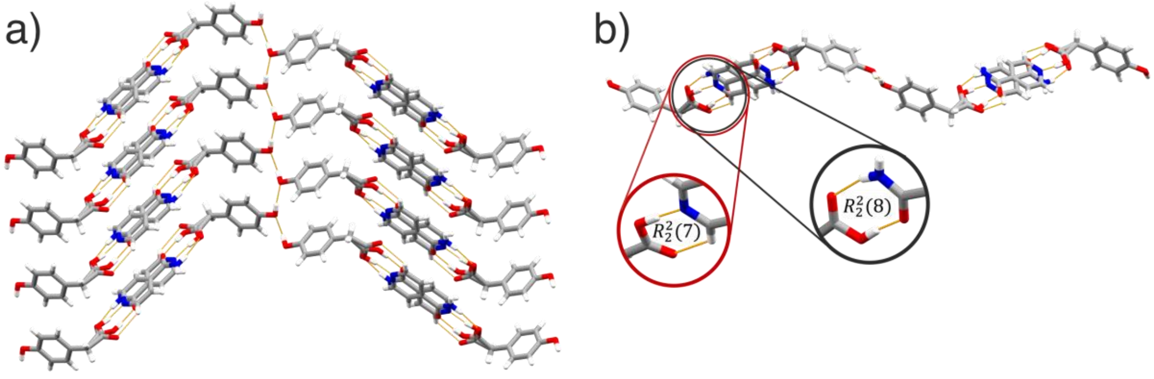

Figure SI.3. Single crystal X-ray structure of a two-dimensional molecular INCT-HPAA sheet viewed: a) along the crystallographic axis *a*, and b) along the crystallographic axis *b*.

I.V INCT-HPPA 2:1

INCT and HPAA co-crystallise in the triclinic space group $P\overline{1}$ with two molecules of INCT and one molecule of HPAA in the asymmetric unit. The INCT molecules form dimers that are sustained by N–H(amide)···O(amide) hydrogen bonds that form $R_2^2(8)$ patterns (Figure SI.4a). Each INCT dimer is hydrogen-bonded to another INCT dimer through another set of N–H(amide)···O(amide) hydrogen bonds to form a tetramer, which exhibits an $R_4^2(8)$ hydrogen-bond pattern (Figure SI.4a). The resulting tetramers are linked into a flat, two-dimensional, hydrogen-bonded molecular sheet through HPAA molecules (Figure SI.4b). These sheets are sustained by O–H(carboxyl)···N(pyridyl), O–H(hydroxyl)···N(pyridyl) and N–H(amide)···O(carboxyl) hydrogen bonds (all defining $D(2)$ patterns), as well as weaker C–H···O and C–H···π interactions. The sheets stack then form a three-dimensional structure that is held together by C–H···O and C–H···N interactions.

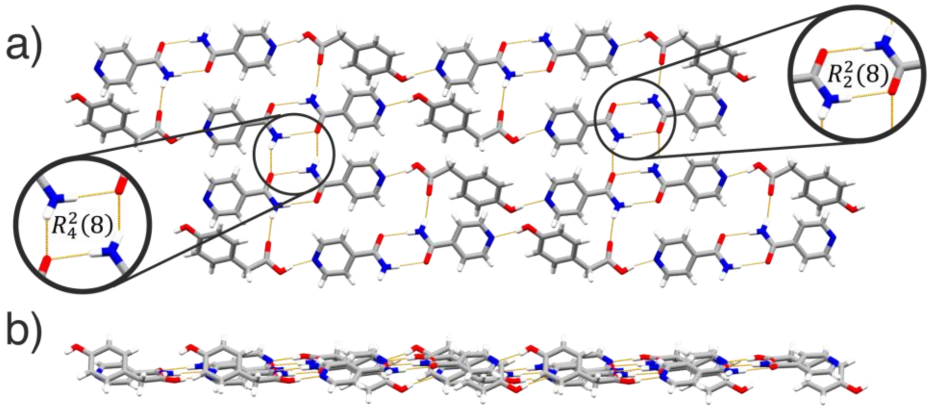

Figure SI.4. Single crystal X-ray structure of a two-dimensional molecular INCT-HPAA sheet viewed: a) along the crystallographic planes (10$\overline{1}$) and (1$\overline{3}$1), and b) along the crystallographic planes (102) and (110).

I.VI INCT-DHPPA 1:1

INCT and DHPAA co-crystallise in the triclinic space group $P\overline{1}$ with both one molecule of INCT and DHPAA in the asymmetric unit. The INCT and two DHPAA molecules form molecular ribbons that are characterised by two types of cyclic substructures (Figure SI.5). The first one exhibits an $R_4^4(22)$ pattern that is established by $D(2)$ O–H(hydroxyl)···N(pyridyl) and $D(2)$ N–H(amide)···O(carboxyl) hydrogen bonds, while the second one exhibits a $R_4^4(26)$ pattern that is defined by $D(2)$ N–H(amide)···O(carboxyl) and $D(2)$ O–H(hydroxyl)···O(amide) hydrogen bonds. The molecular ribbons are stacked and held together by $D(2)$ O–H(hydroxyl)···O(amide) hydrogen bonds, which account for a third type of cyclic hydrogen pattern, namely a $R_4^4(24)$ motif. The molecular sheets stack and the resulting three-dimensional structure is held together by C–H···O and π···π interactions.

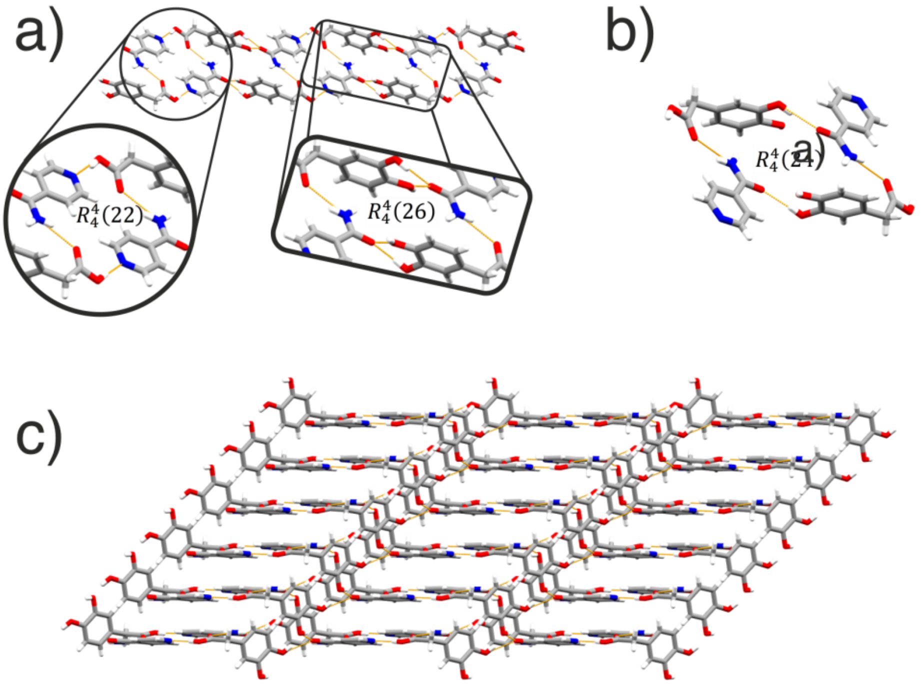

Figure SI.5. a) Single crystal X-ray structure of a two-dimensional molecular INCT-DHPAA ribbon viewed along the crystallographic axis *a*; b) a perspective view of a set of hydrogen bonds that links two stacked molecular ribbons, and c) a view of the two-dimensional INCT-DHPAA sheet viewed along the crystallographic planes (110) and (001).

II. Additional data

Table SII.1. Starting models used for Rietveld refinement against diffraction patterns collected for an equimolar mixture of INCT and HBA.

Species	Model
INCT *form I*	EHOWIH01*
INCT *form II*	EHOWIH02*
HBA	JOZZIH*
Co-crystal *form I*	VAKTOR*
Co-crystal *form II*	This work

*taken from the CSD.

Table SII.2. Refined unit cell parameters for a physical mixture of INCT and HBA at 40 °C. The starting models were taken from the CSD (INCT: EHOWIH01; HBA: JOZZIH).

Property	INCT<br>*Form I*	HBA	Co-crystal<br>*This work*
T / °C		40	
Space group	$P2_1/c$	$P2_1/a$	$P2_1/c$
a / Å	10.2442(8)	18.504(4)	10.656(9)
b / Å	5.7426(5)	5.2373(3)	29.31(12)
c / Å	10.0650(7)	6.3345(13)	7.743(7)
α / °	90	90	90
β / °	97.259(7)	93.205(12)	97.82(10)
γ / °	90	90	90
Cell volume / Å^3	587.36(8)	612.95(16)	2396(10)
R_{wp}		0.0757	
$R_{wp\text{-}bkd}$		0.1843	
Phase Fraction*	43.1 %	50.5 %	5.3 %

*Due to the graininess of the sample, representative errors cannot be calculated. However, the authors conservatively estimate the error to be in the region of 5 %. As a result the sum does not equal 100 % as a small portion of the material was assigned to the other structures included in the refinement.

Table SII.3. Refined unit cell parameters for a physical mixture of INCT and HBA at 97 °C. The starting models for the raw materials were taken from the CSD (INCT: EHOWIH01; HBA: JOZZIH; Co-crystal: VAKTOR) and single crystal data reported in Table SI.1 was used for the new co-crystal.

Property	INCT *Form I*	HBA	Co-crystal *VAKTOR*	Co-crystal *This work*
T / °C			97	
Space group	$P2_1/c$	$P2_1/a$	$P2_1/n$	$P2_1/c$
a / Å	10.2815(12)	18.601(4)	5.964(7)	10.722(4)
b / Å	5.7575(6)	5.2598(4)	9.536(10)	29.75(4)
c / Å	10.0893(9)	6.3409(16)	21.253(19)	7.725(4)
α / °	90	90	90	90
β / °	96.909(9)	92.837(1)	98.78(9)	98.90(6)
γ / °	90	90	90	90
Cell volume / Å^3	592.91(11)	619.6(2)	1194(2)	2427(3)
R_{wp}			0.0924	
$R_{wp\text{-}bkd}$			0.2227	
Phase Fraction*	37.6 %	50.37 %	5.85 %	6.10 %

*Due to the graininess of the sample, representative errors cannot be calculated. However, the authors conservatively estimate the error to be in the region of 5 %.

Table SII.4. Refined unit cell parameters for a physical mixture of INCT and HBA at 122 °C. The starting models for the raw materials were taken from the CSD (INCT I: EHOWIH01; INCT II: EHOWIH02; HBA: JOZZIH; reported co-crystal: VAKTOR), and the single crystal data reported in Table SI.1 were used for the new co-crystal structure.

Property	INCT *Form I*	HBA	INCT *Form II*	Co-crystal *VAKTOR*	Co-crystal *This work*
T / °C			122		
Space group	$P2_1/c$	$P2_1/a$	$P2_1/c$	$P2_1$/n	$P2_1/c$
a / Å	10.306(1)	18.653(5)	16.267(6)	6.040(4)	10.628(6)
b / Å	5.7654(7)	5.2696(4)	7.996(7)	9.560(6)	29.18(7)
c / Å	10.107(1)	6.346(2)	9.922(8)	21.16(1)	7.826(5)
α / °	90	90	90	90	90
β / °	96.82(1)	92.69(2)	105.66(6)	96.39(6)	98.26(8)
γ / °	90	90	90	90	90
Cell volume / Å^3	596.29(12)	623.1(3)	1242(1)	1214(1)	2402(6)
R_{wp}			0.0923		
$R_{wp\text{-}bkd}$			0.2191		
Phase Fraction*	31.4 %	41.5 %	7.3 %	10.7 %	9.1 %

*Due to the graininess of the sample, representative errors cannot be calculated. However, the authors conservatively estimate the error to be in the region of 5 %.

Table SII.5. Refined unit cell parameters for a physical mixture of INCT and HBA at 139 °C. The starting models for the raw materials were taken from the CSD (INCT II: EHOWIH02; HBA: JOZZIH; reported co-crystal: VAKTOR).

Property	HBA	INCT *Form II*	Co-crystal *VAKTOR*
T / °C		139	
Space group	$P2_1/a$	$P2_1/c$	$P2_1/n$
a / Å	18.667(5)	16.298(1)	6.060(1)
b / Å	5.2744(4)	7.993(1)	9.547(2)
c / Å	6.354(2)	9.964(2)	21.279(4)
α / °	90	90	90
β / °	92.59(2)	105.29(1)	96.26(2)
γ / °	90	90	90
Cell volume / Å^3	625.0(3)	1252.0(3)	1223.8(4)
R_{wp}		0.0930	
$R_{wp\text{-}bkd}$		0.2280	
Phase Fraction*	38.1 %	35.3 %	26.6 %

*Due to the graininess of the sample, representative errors cannot be calculated. However, the authors conservatively estimate the error to be in the region of 5 %.

Table SII.6. Refined unit cell parameters for a physical mixture of INCT and HBA at 170 °C. The starting models were taken from the CSD (HBA: JOZZIH; co-crystal: VAKTOR).

Property	HBA	INCT *Form II*	Co-crystal *VAKTOR*
T / °C		170	
Space group	$P2_1/a$	$P2_1/c$	$P2_1/n$
a / Å	18.780(11)	16.450(9)	6.064(1)
b / Å	5.2887(9)	8.070(9)	9.571(1)
c / Å	6.381(5)	9.905(11)	21.377(2)
α / °	90	90	90
β / °	92.61(4)	105.43(9)	96.442(11)
γ / °	90	90	90
Cell volume / Å^3	633.0(6)	1268(2)	1232.805(11)
R_{wp}		0.0855	
$R_{wp\text{-}bkd}$		0.2821	
Phase Fraction*	9.6 %	4.8 %	73.8 %

*Due to the graininess of the sample, representative errors cannot be calculated. However, the authors conservatively estimate the error to be in the region of 5 %. As a result the sum does not equal 100 % as a small portion of the material was assigned to the other structures included in the refinement.

Table SII.7. Refined unit cell parameters for a physical mixture of INCT and HPAA at 40 °C. The starting models for the raw materials were taken from the CSD (INCT: EHOWIH01; HPAA: QAPBAL) and single crystal data reported in Table SI.1 was used for the co-crystal.

Property	INCT *Form I*	HPAA	INCT-HPAA *Co-crystal*
T / °C		40	
Space group	$P2_1/c$	$P2_12_12_1$	*P*-1
a / Å	10.251(2)	5.3187(9)	6.792(6)
b / Å	5.729(1)	9.027(3)	12.31(2)
c / Å	10.065(1)	15.443(7)	12.81(2)
α / °	90	90	111.08(9)
β / °	97.30(1)	90	95.80(9)
γ / °	90	90	99.36(7)
Cell volume / Å^3	586.24(15)	741.4(4)	971(2)
R_{wp}		0.1431	
$R_{wp\text{-}bkd}$		0.2893	
Phase Fraction*	61.8 %	25.0 %	13.2 %

*Due to the graininess of the sample, representative errors cannot be calculated. However, the authors conservatively estimate the error to be in the region of 5 %.

Table SII.8. Refined unit cell parameters for a mixture of INCT and HPAA at 113 °C. The starting models for the raw materials were taken from the CSD (INCT: EHOWIH01; HPAA: QAPBAL) and single crystal data reported in Table SI.1 used for the co-crystal.

Property	INCT *Form I*	HPAA	INCT-HPAA *Co-crystal*
T / °C		113	
Space group	$P2_1/c$	$P2_12_12_1$	*P*-1
a / Å	10.284(2)	5.24(4)	6.8176(8)
b / Å	5.762(10)	9.14(6)	12.3148(19)
c / Å	10.0956(10)	15.27(9)	12.9137(19)
α / °	90	90	110.754(10)
β / °	96.815(11)	90	95.425(11)
γ / °	90	90	99.067(8)
Cell volume / Å^3	594.0(3)	731(8)	987.8(3)
R_{wp}		0.0678	
$R_{wp\text{-}bkd}$		0.1810	
Phase Fraction*	34.0 %	0.8 %	65.2 %

*Due to the graininess of the sample, representative errors cannot be calculated. However, the authors conservatively estimate the error to be in the region of 5 %.

Table SII.9. Refined unit cell parameters for a mixture of INCT and DHPAA at 40 °C. The starting models for the raw materials were taken from the CSD (INCT: EHOWIH01; DHPAA: NELTON).

Property	INCT *Form I*	DHPAA
T / °C	40	
Space group	$P2_1/c$	*Pbca*
a / Å	10.2414(7)	16.1356(19)
b / Å	5.7407(4)	11.5933(8)
c / Å	10.0693(5)	7.9176(3)
α / °	90	90
β / °	97.253(5)	90
γ / °	90	90
Cell volume / Å^3	587.27(6)	1481.1(2)
R_{wp}	0.0556	
$R_{wp\text{-}bkd}$	0.1693	
Phase Fraction*	54.5 %	45.5 %

*Due to the graininess of the sample, representative errors cannot be calculated. However, the authors conservatively estimate the error to be in the region of 5 %.

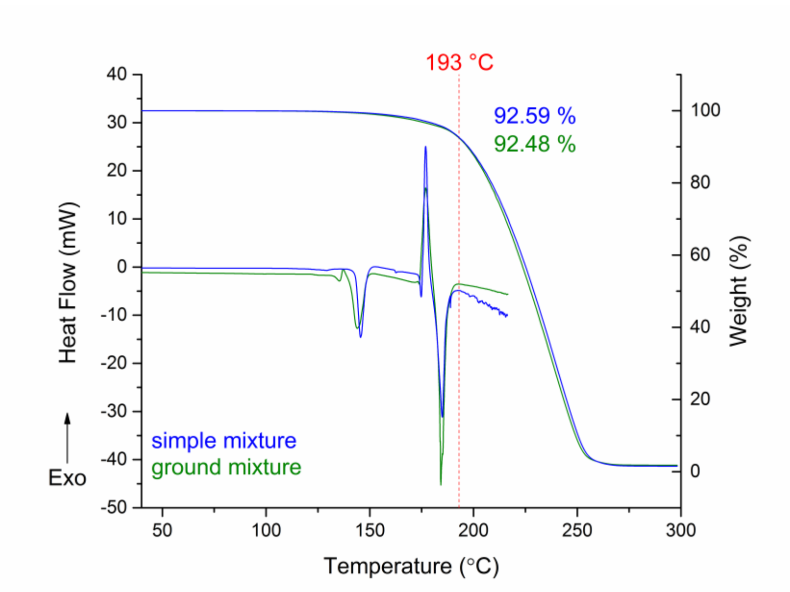

Figure SII.1. TGA thermograms recorded for a simple equimolar mixture of INCT and HBA and a mixture in which the two constituents had been ground separately in a pestle and mortar prior to mixing.

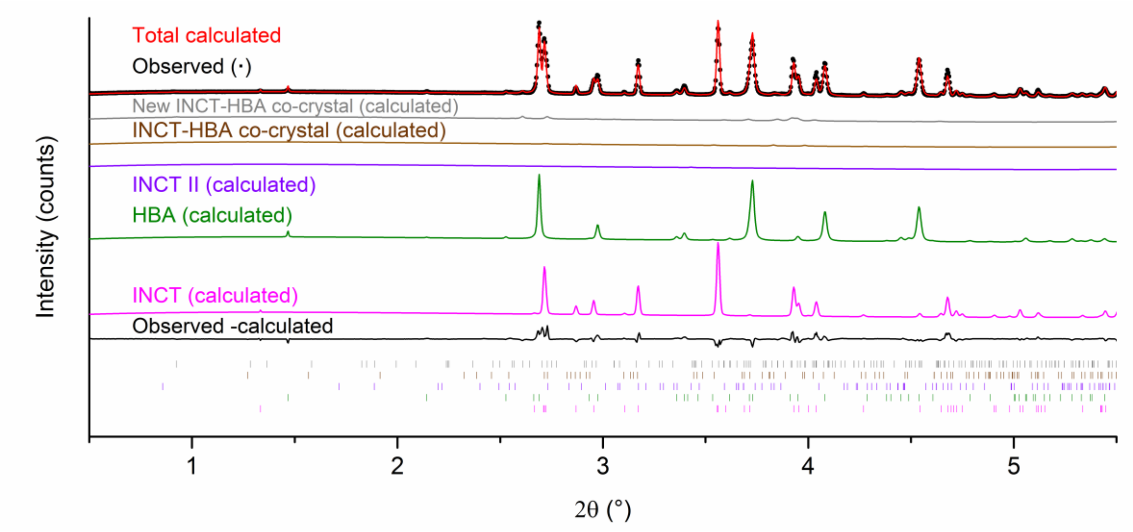

Figure SII.2. Rietveld refinement data for a physical mixture of INCT and HBA at 40 °C. Tick marks show the positions of allowed reflections of each phase.

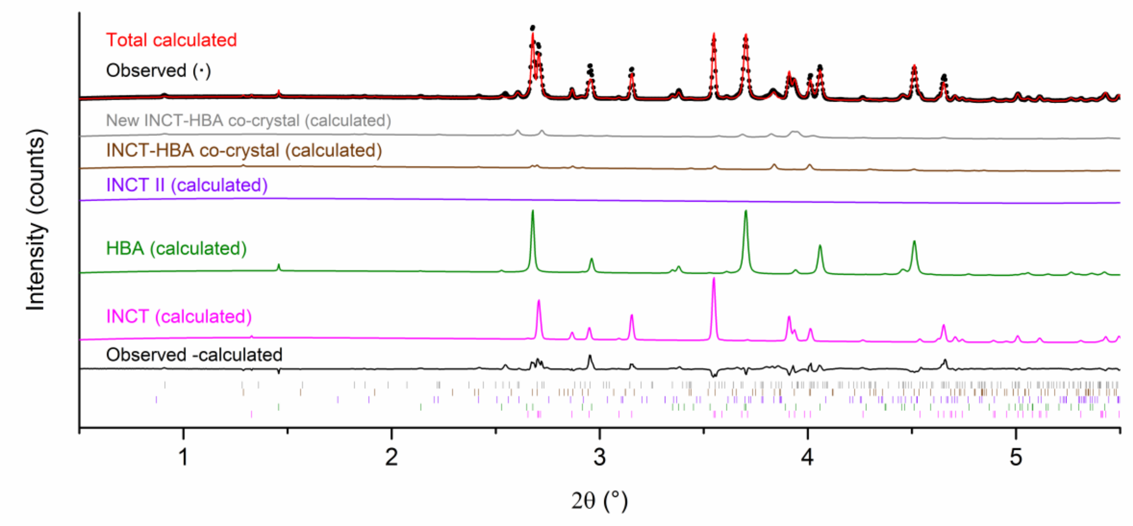

Figure SII.3. Rietveld refinement data for a physical mixture of INCT and HBA at 97 °C. Tick marks show the positions of allowed reflections of each phase.

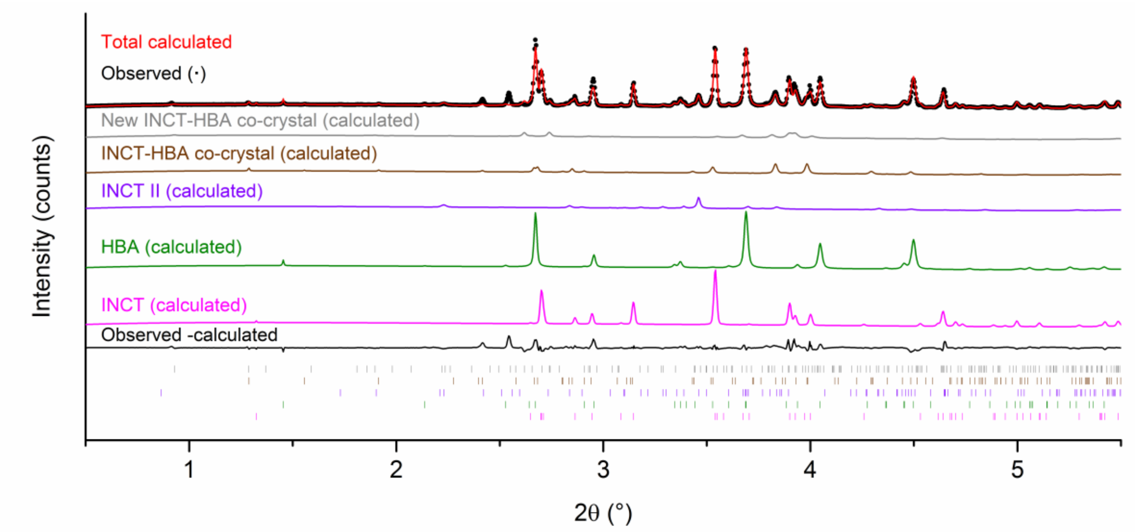

Figure SII.4. Rietveld refinement data for a physical mixture of INCT and HBA at 122 °C. Tick marks show the positions of allowed reflections of each phase.

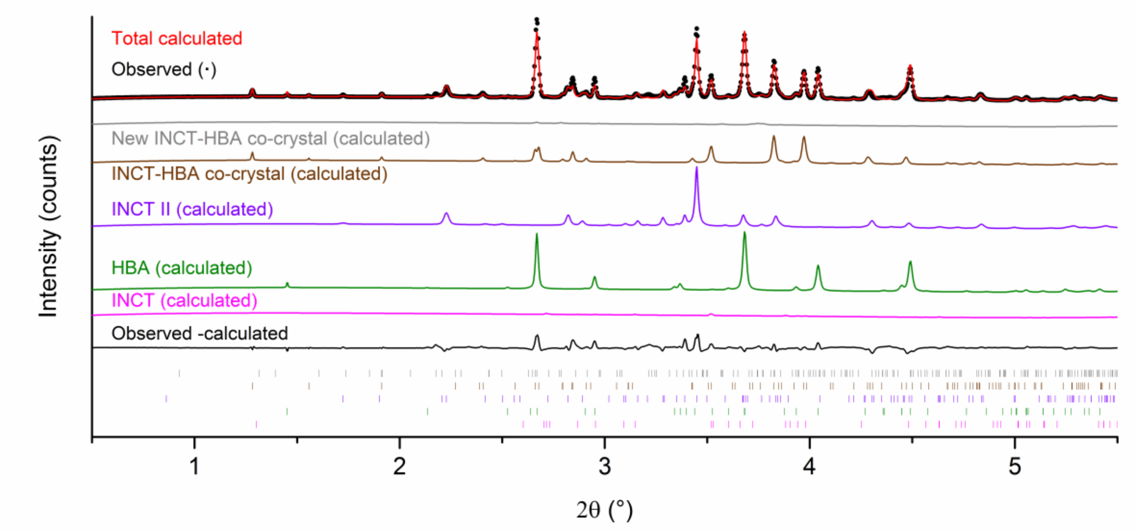

Figure SII.5. Rietveld refinement data for a physical mixture of INCT and HBA at 139 °C. Tick marks show the positions of allowed reflections of each phase.

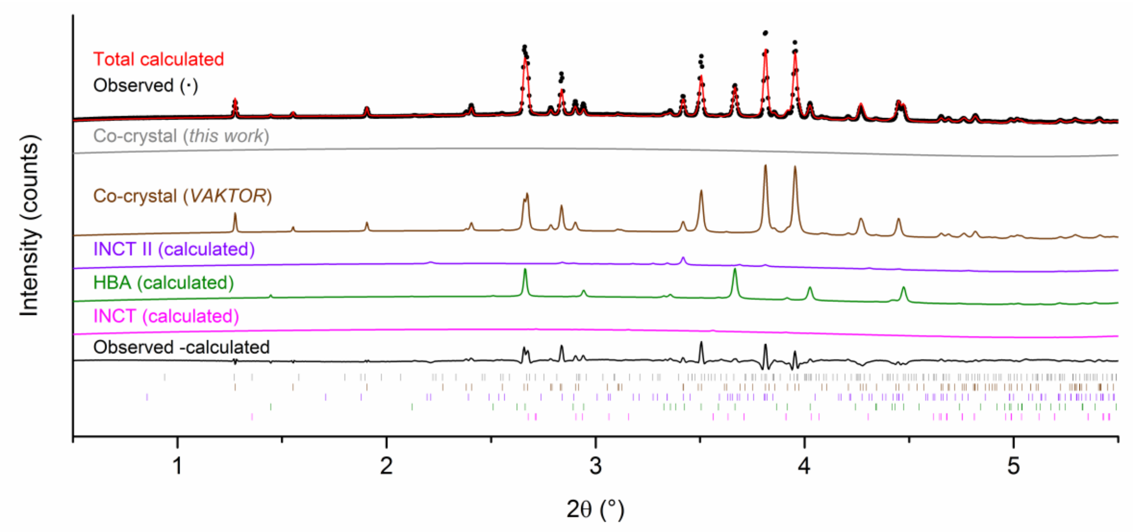

Figure SII.6. Rietveld refinement data for a physical mixture of INCT and HBA at 170 °C. Tick marks show the positions of allowed reflections of each phase.

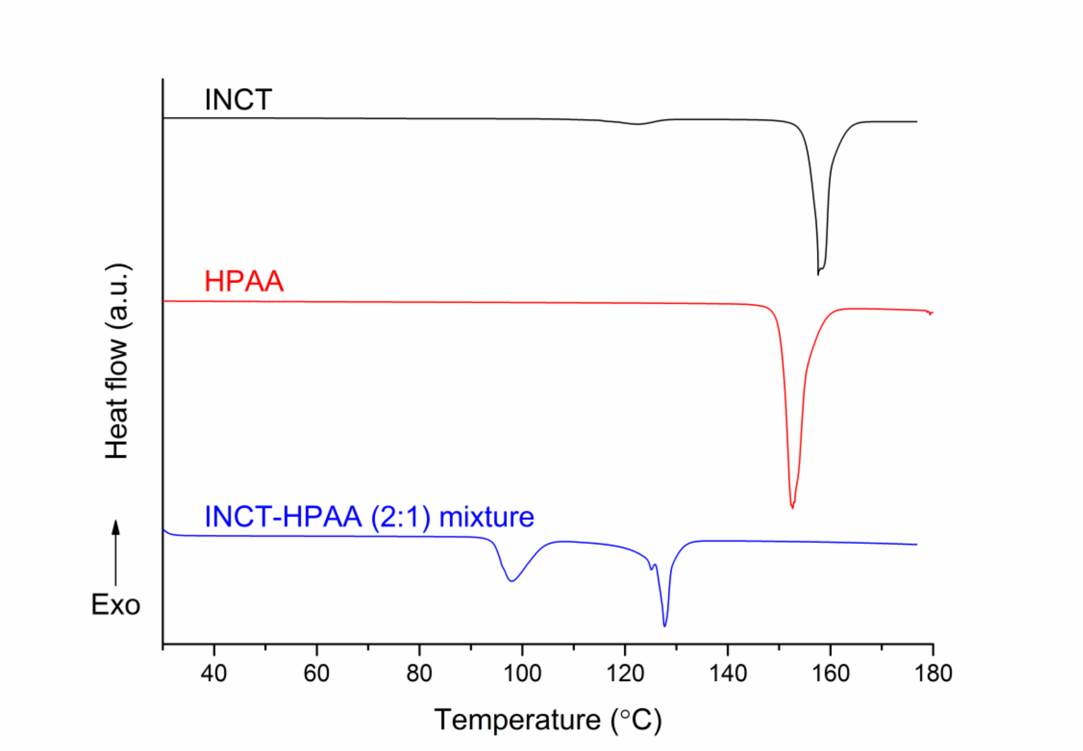

Figure SII.7. DSC thermograms recorded for INCT (black), HPAA (red), and a mixture of the two raw materials (2:1 molar ratio) as received (blue).

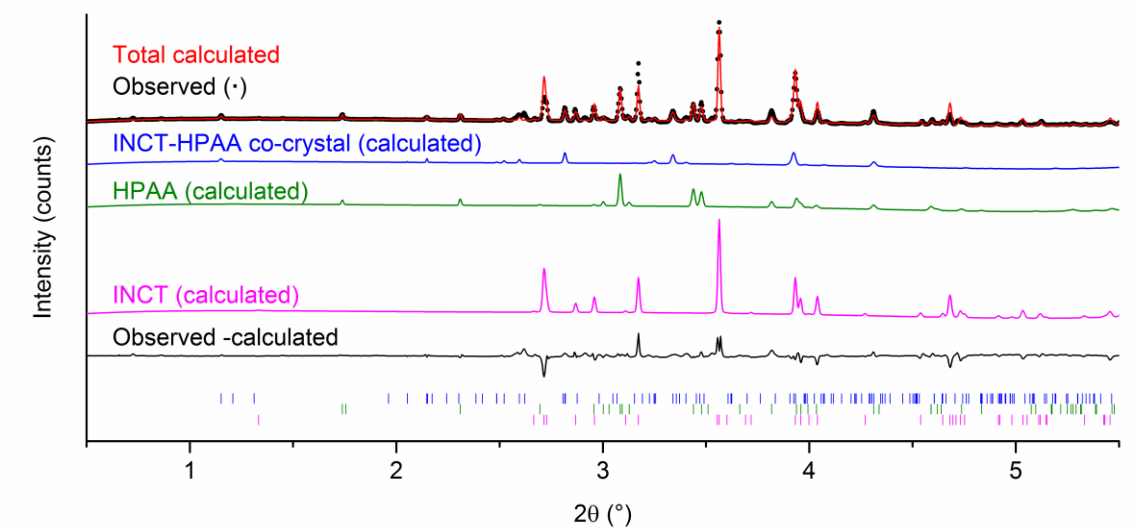

Figure SII.8. Rietveld refinement data for a mixture of INCT and HPAA at 40 °C. Tick marks show the positions of allowed reflections of each phase.

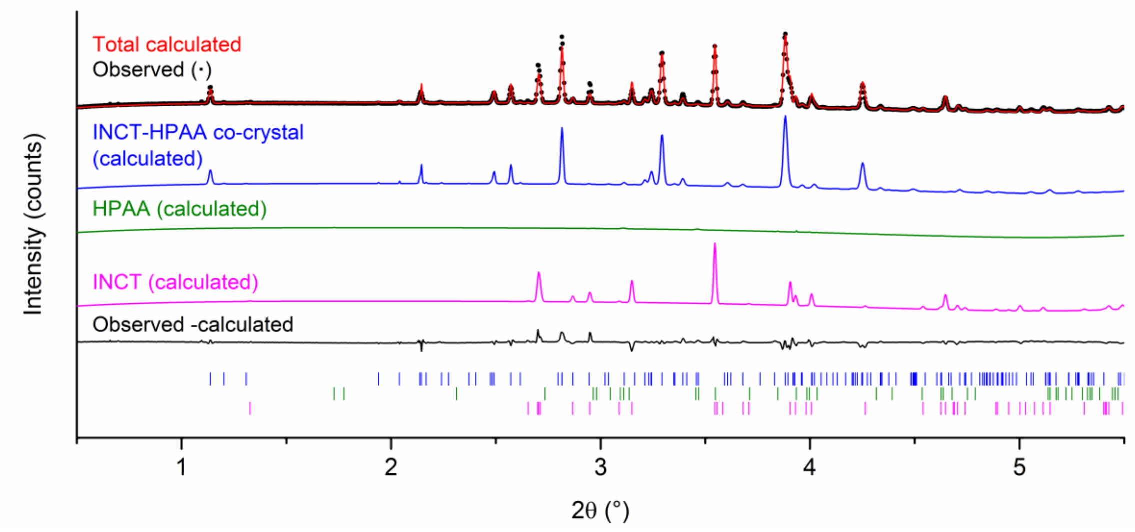

Figure SII.9. Rietveld refinement data for a mixture of INCT and HPAA at 113 °C. Tick marks show the positions of allowed reflections of each phase.

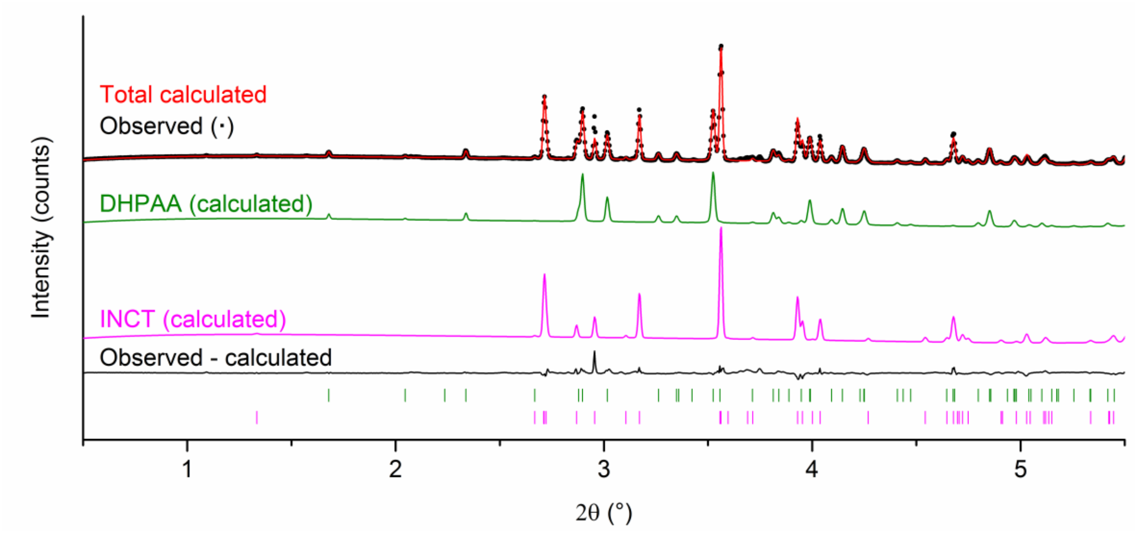

Figure SII.10. Rietveld refinement data for a mixture of INCT and DHPAA at 40 °C. Tick marks show the positions of allowed reflections of each phase.

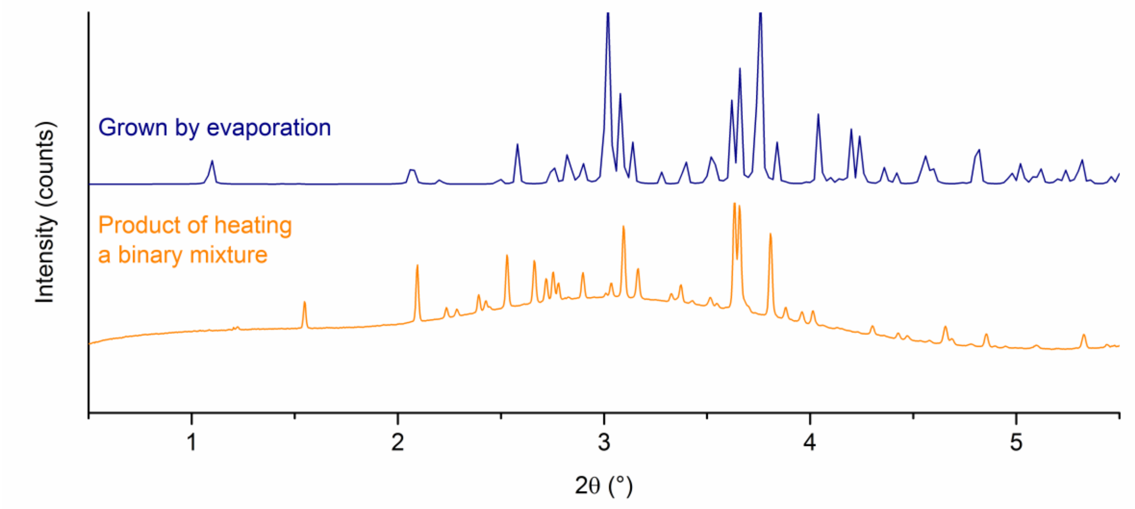

Figure SII.11. Powder diffraction pattern for crystals produced by heating an equimolar binary mixture of INCT and DHPAA to 122 °C and the predicted pattern for crystals grown by solvent evaporation from an equimolar ethanolic solution.

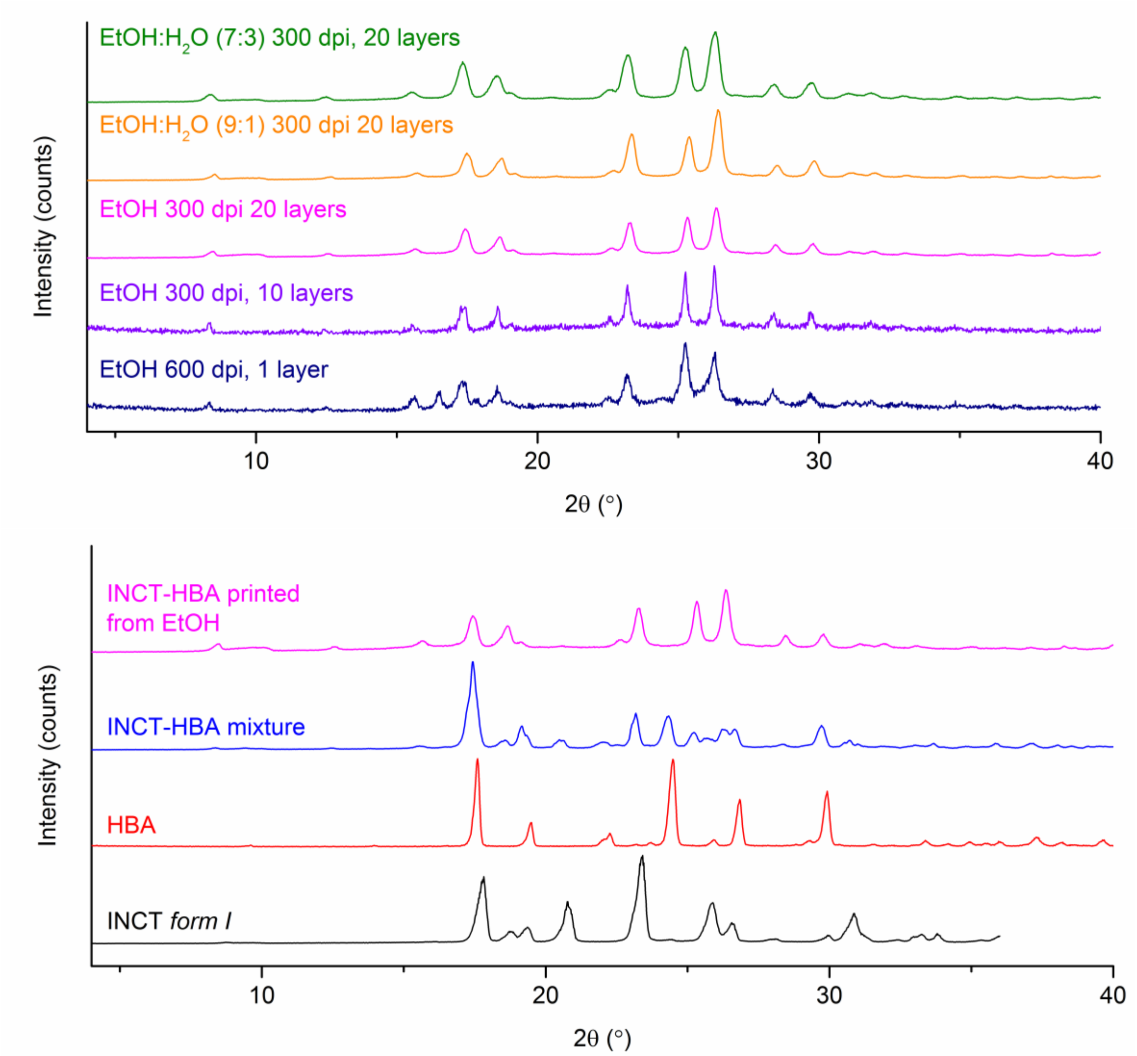

Figure SII.12. Diffraction patterns for crystals printed from an equimolar ethanolic solution of ICNT and HBA. Top: the effect of varying the solvent system used and the print parameters. Bottom: a comparison of the patterns of INCT I, HBA, a physical mixture of the two, and the printed crystals.

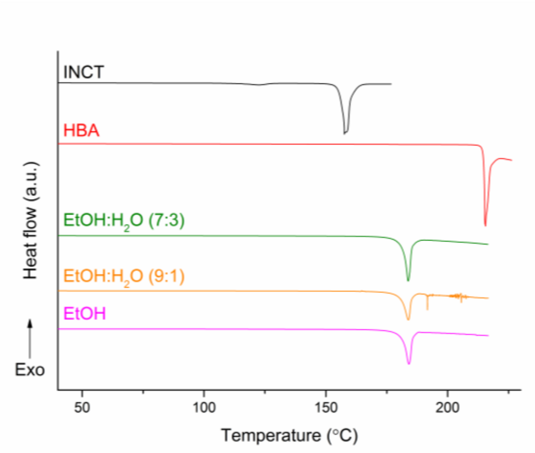

(a)

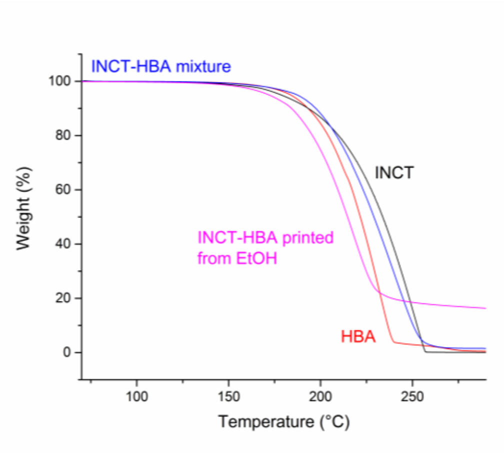

(b)

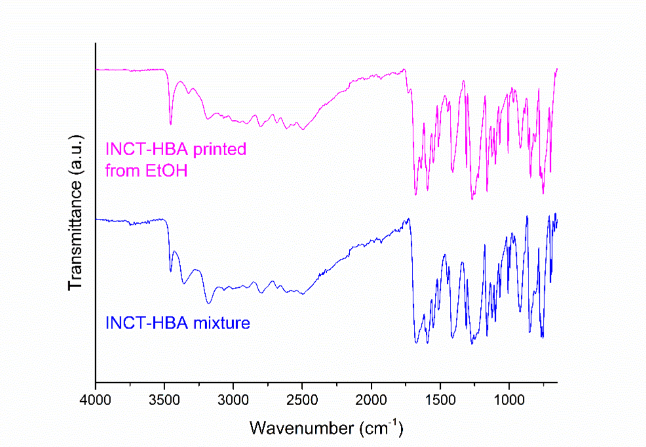

(c)

Figure SII.13. Characterising data on INCT-HBA co-crystals prepared by inkjet printing. **(a) DSC data.** All three of the printed mixtures show a single melting endotherm with an onset at 181-182 °C followed by a steady drop in heat flow caused by decomposition above 190 °C. The raw materials exhibit endotherms with onsets at the melting points of each of the two components (157 °C, INCT II; 215 °C, HBA), with an additional small endotherm (onset 115 °C) in the INCT data representing a conversion from polymorph I to polymorph II. **(b) TGA traces** show that decomposition of the printed crystals begins at a lower temperature than either of the individual components, and also that of a physical mixture of the two. It also levels off at around 20 % mass rather than continuing to decay to 0 %, suggesting that printing has resulted in some kind of interaction between the co-formers. **(c) IR spectra**, revealing significant shift of the N—H stretch at 3360 cm^{-1} in INCT to 3455 cm^{-1} in the printed crystals, which occurs with a concomitant reduction in intensity of the second N—H stretch in INCT (3180 cm^{-1}) and the broad strong O—H stretch seen at 3360 cm^{-1} in the HBA spectrum. The rest of the spectrum of the printed crystals displays all of the peaks associated with the raw materials, as would be expected, with the exception of the region between 1200 cm^{-1} and 1300 cm^{-1} where there is a slight shift to a lower wavenumber of the band relating to the C—O bond of the carbonyl of HBA.

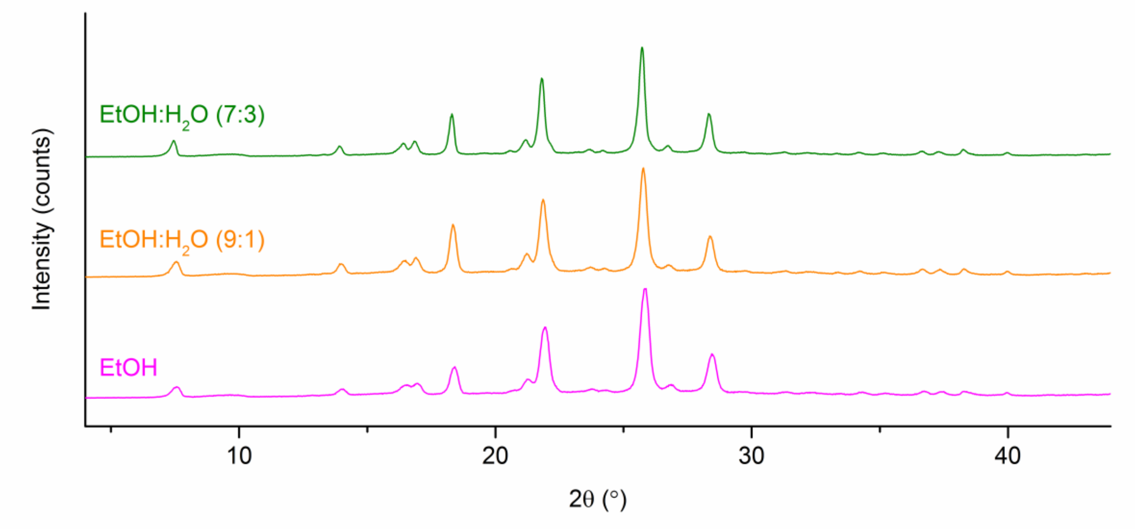

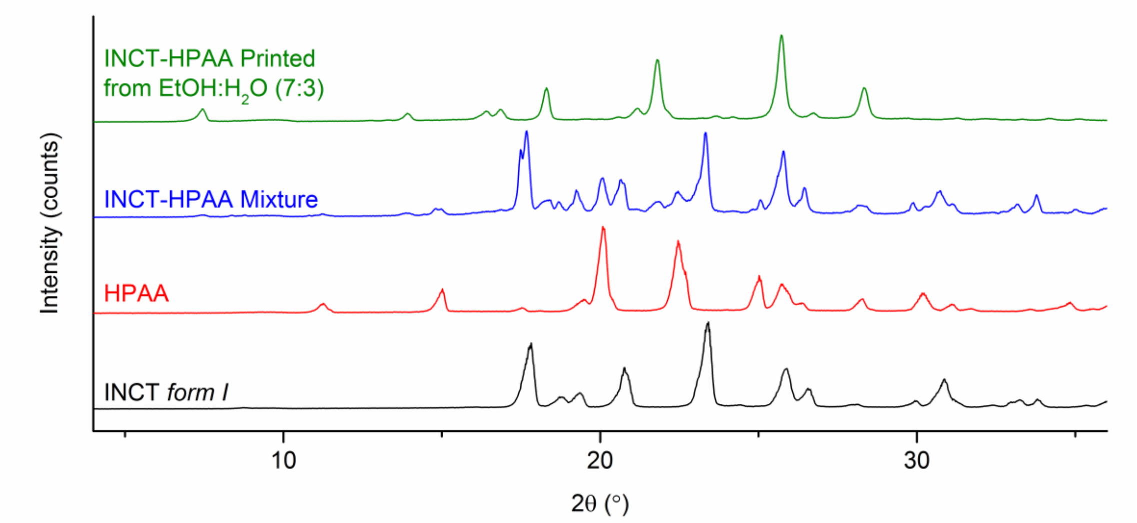

Figure SII.14. Powder diffraction patterns for crystals printed from ethanolic solutions of INCT and HPAA at a molar ratio of 2:1. Top: the effect of varying the solvent system used and the print parameters. Bottom: a comparison of the patterns of the individual coformers, a physical mixture of the two, and the printed crystals.

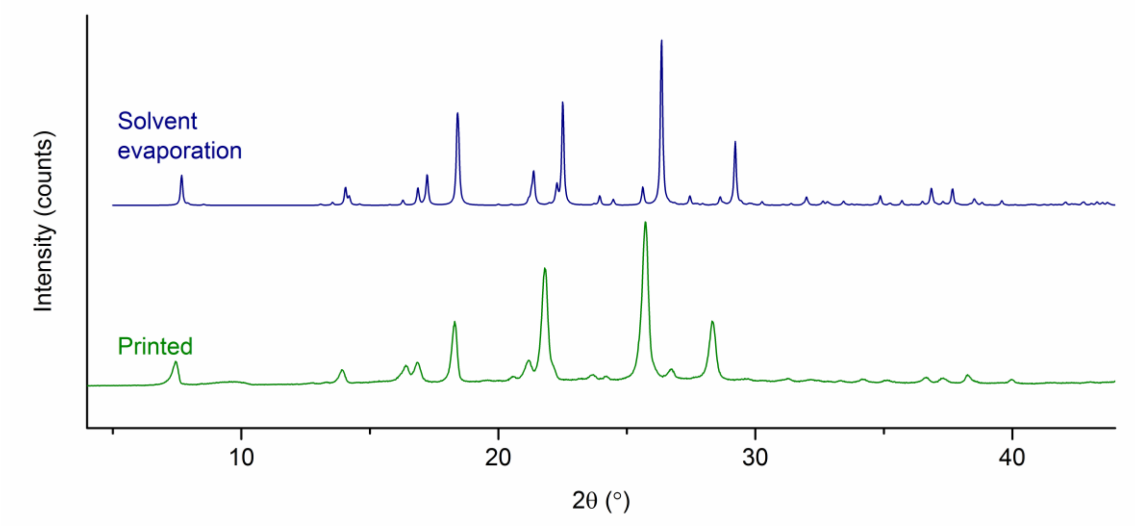

Figure SII.15. Powder diffraction pattern for crystals printed from a solution of INCT:HPAA (2:1 molar ratio) in EtOH:H_2O (7:3) and a predicted pattern for the 2:1 crystals grown from the same solution by solvent evaporation.

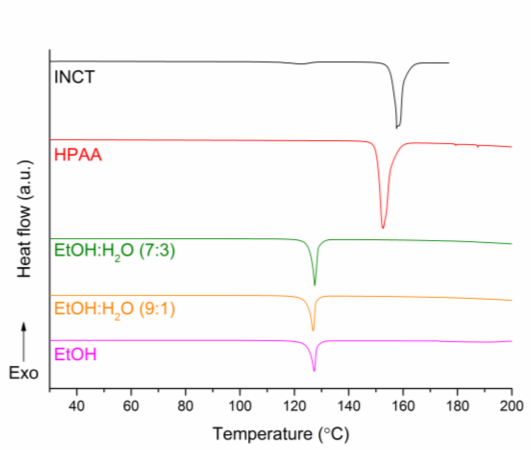

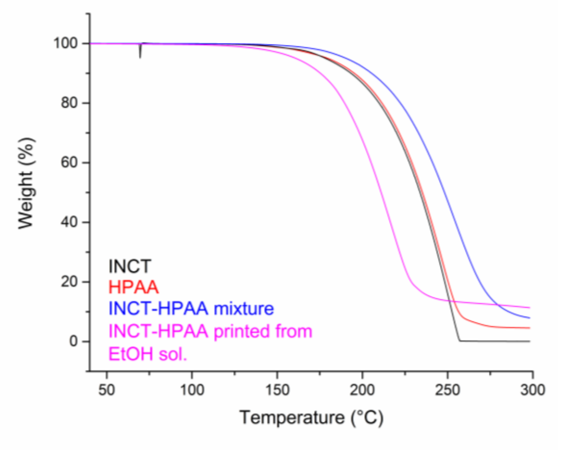

(a) **(b)**

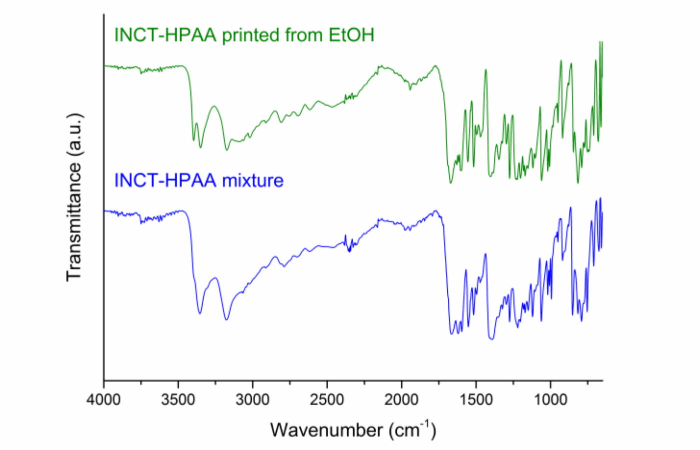

(c)

Figure SII.16. Characterising data on the INCT-HPAA co-crystals prepared by inkjet printing. **(a) DSC data.** All three of the printed crystal thermograms have only one endothermic melting event with an onset at 125-126 °C and an enthalpy of *ca.* 150 J g^{-1}. This event is the melting of the co-crystal formed between the two components. The highest temperature melting endotherms for INCT and HPAA have onsets at 157 °C and 150 °C and have associated enthalpies of fusion of 184 J g^{-1} and 194 J g^{-1} respectively. **(b) TGA traces** revealing that the printed crystals begin to lose mass at temperatures 25-30 °C below that recorded for either of the crystalline components individually or in a physical mixture at the same ratio as used for printing and solvent evaporation. **(c) IR spectra**, showing band shifts. INCT has two strong bands at 3355 cm^{-1} and 3175 cm^{-1} characteristic of stretching of N—H bonds in a primary amide. HPAA has a strong band at 3245 cm^{-1} resulting from the stretching of the O—H bond of the alcohol in the O1 position and a much broader medium intensity band peaking at 2990 cm^{-1} relating to the stretching of the carboxylic acid O—H bond. The N—H bands of the INCT have not shifted, but the bands relating to the O—H bonds in HPAA have moved to higher wavenumbers by 150 cm^{-1} and 110 cm^{-1} respectively.

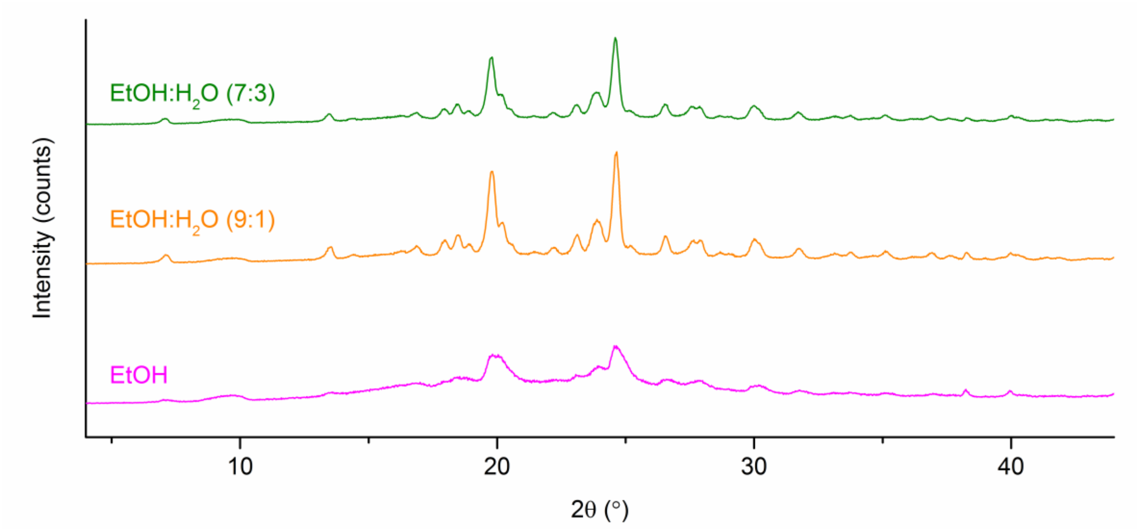

Figure SII.17. Diffraction patterns for crystals printed from equimolar solutions of INCT and DHPAA in EtOH, EtOH with 10 % v/v water and EtOH with 30 % v/v water.

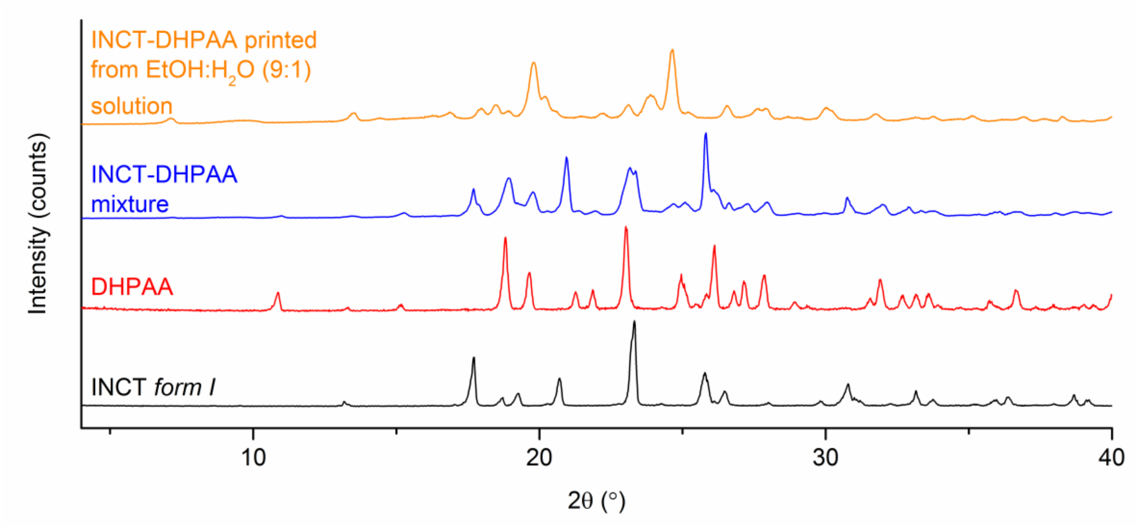

Figure SII.18. Diffraction patterns for INCT, DHPAA, a physical mixture of the two and crystals printed from an equimolar solution of the two in ethanol and water (9:1 v/v).

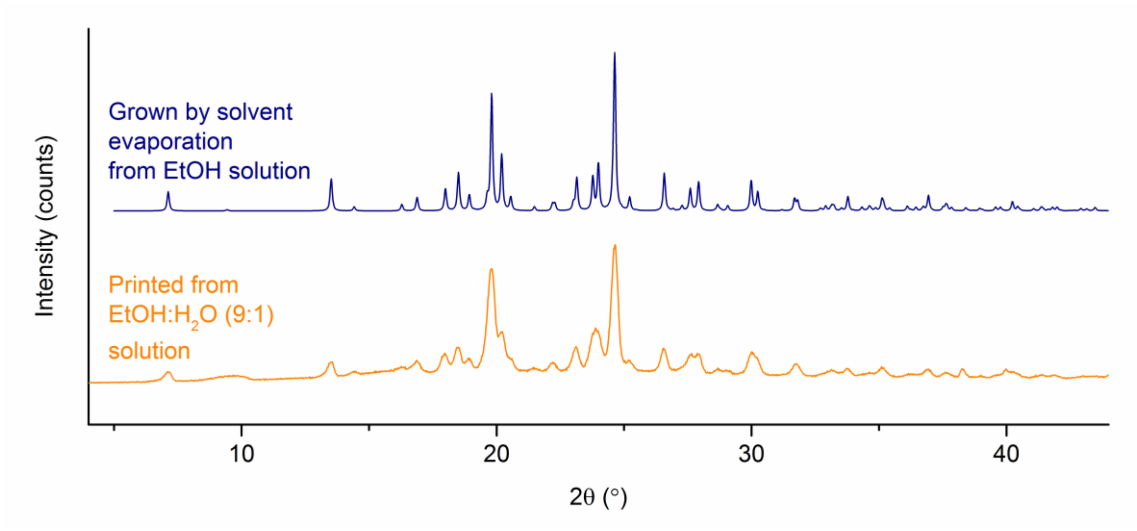

Figure SII.19. Powder diffraction pattern for crystals printed from an equimolar solution of INCT:DHPAA in EtOH:H_2O (9:1 v/v) and the predicted powder pattern for the ICNT-DHPAA co-crystal.

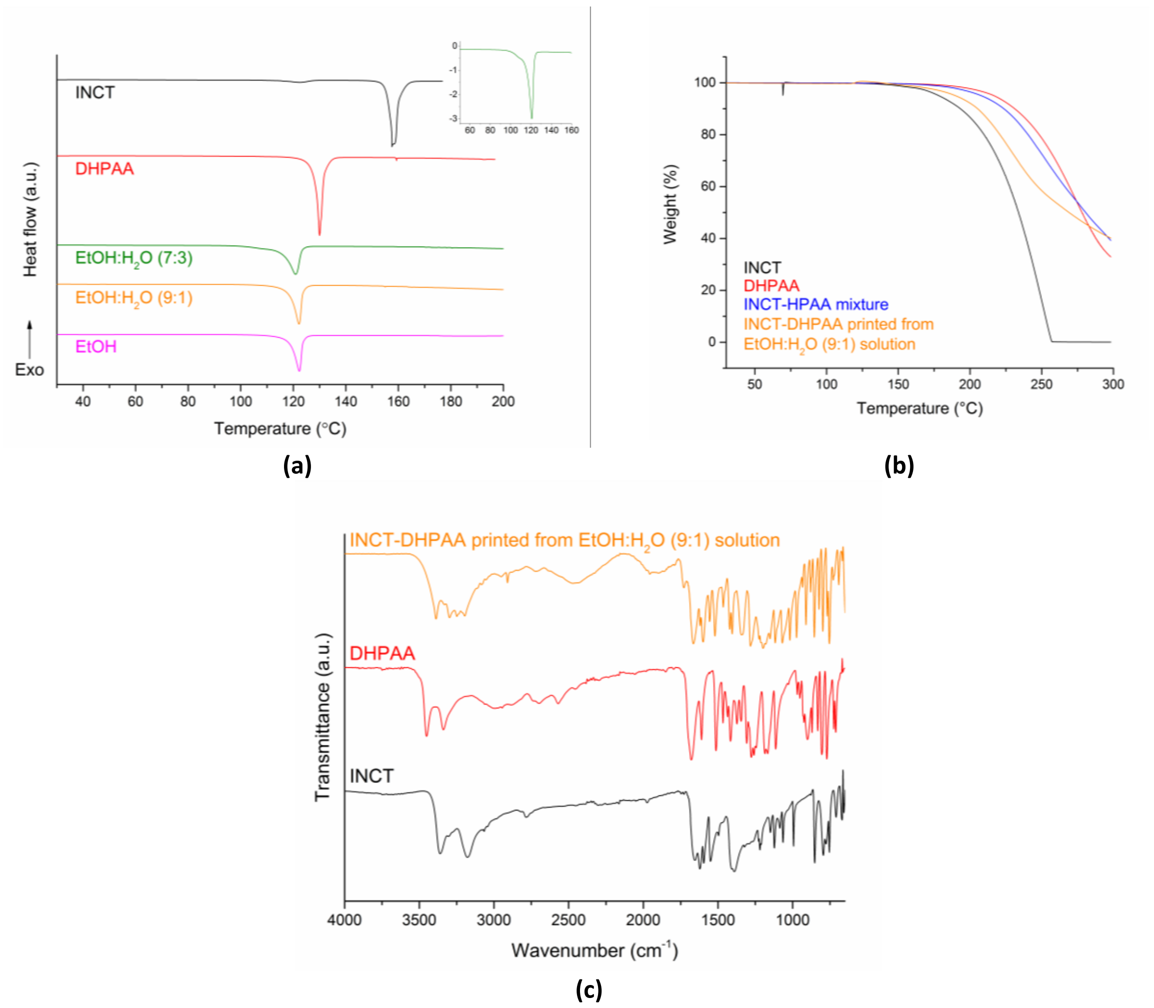

Figure SII.20. Characterising data on the INCT-DHPAA co-crystals prepared by inkjet printing. **(a) DSC thermograms** showing the co-crystal to have a different melting point to the raw materials. The inset plot shows a magnified view of the endothermic events in the trace for the sample printed from EtOH:H_2O (7:3) solution. **(b) TGA traces** revealing that the printed crystals decompose at a temperature higher than INCT but lower than DHPAA or a physical mixture of the two components. **(c) IR spectra** displaying peak shifts.

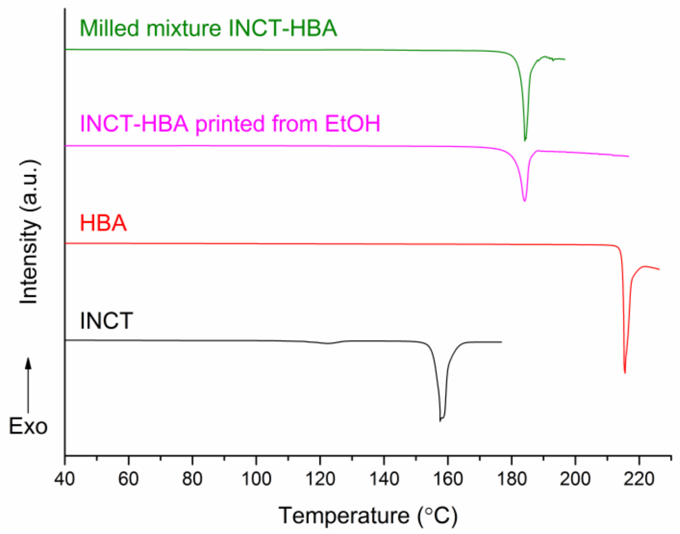

Figure SII.21. DSC data for INCT, HBA, crystals printed from an equimolar ethanolic solution of both and a mixture of the two following ball milling.

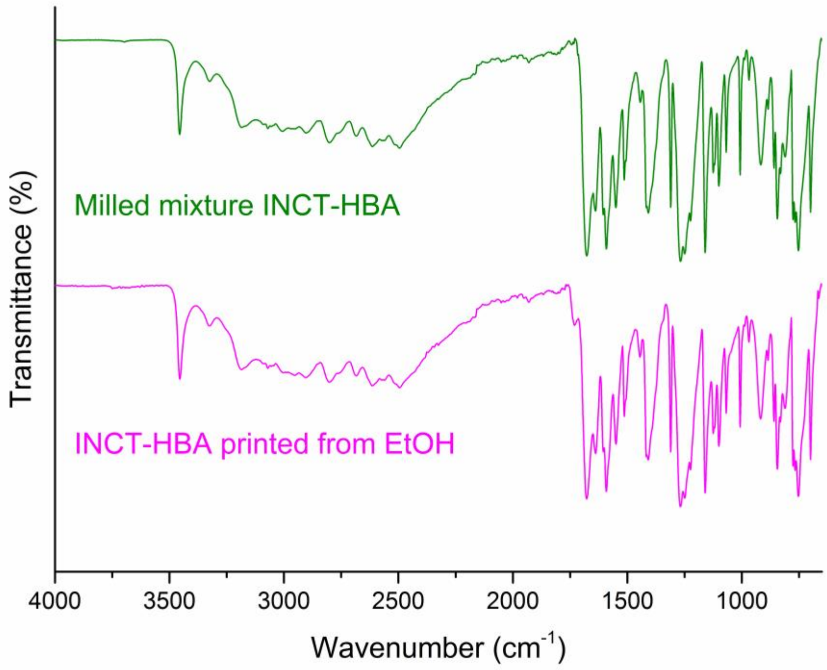

Figure SII.22. FTIR spectra for a ball milled mixture of INCT and HBA and for crystals printed from an equimolar ethanolic solution of the two.

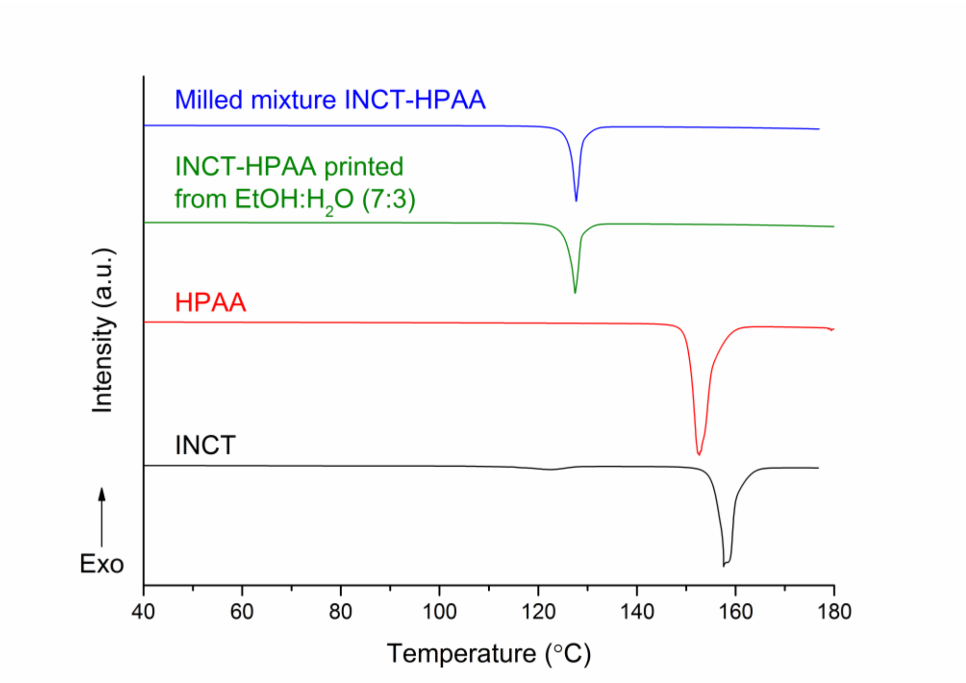

Figure SII.23. DSC data for INCT, HPAA, crystals printed from a 2:1 molar ratio solution of the two in EtOH:H_2O (7:3 v/v), and a 2:1 molar ratio mixture of the two following ball milling.

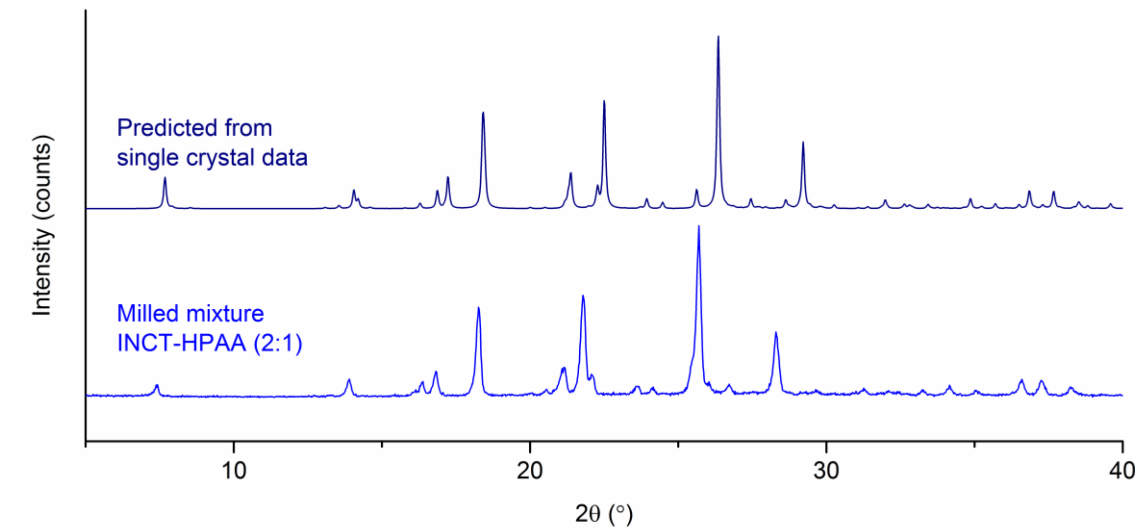

Figure SII.24. PXRD patterns collected for a ball milled mixture of INCT and HPAA and that predicted from single crystal data for the 2:1 co-crystal.

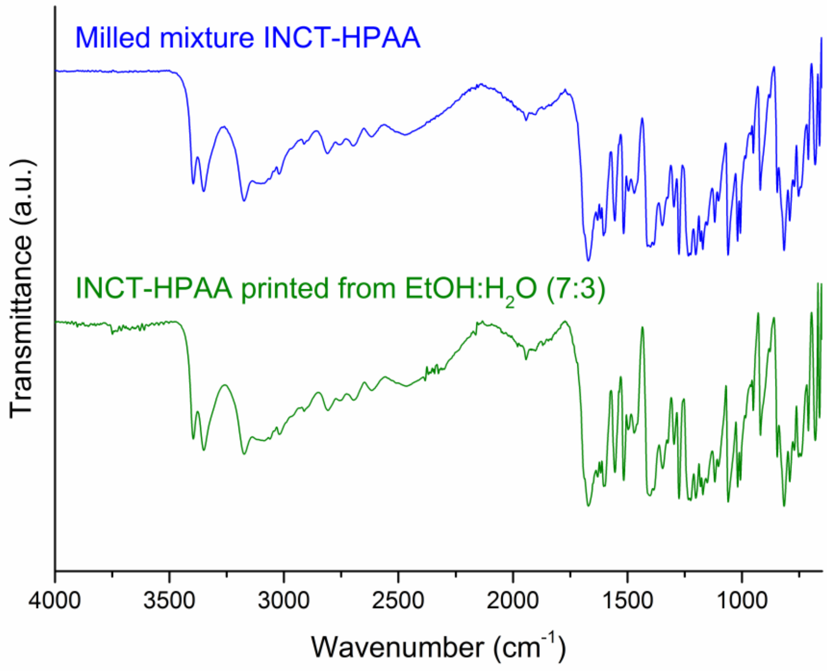

Figure SII.25. FTIR spectra for a ball milled 2:1 molar mixture of INCT and HBA and for crystals printed from a 2:1 molar solution of the two in EtOH:H_2O (7:3).

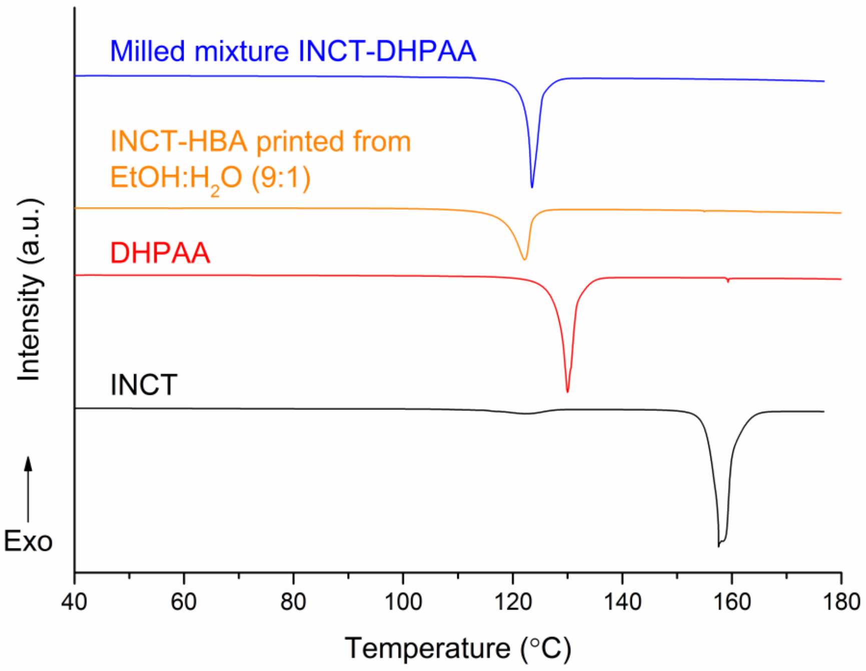

Figure SII.26. DSC data for INCT, DHPAA, crystals printed from an equimolar solution of both in EtOH:H2O (9:1) and a mixture of the two following ball milling.

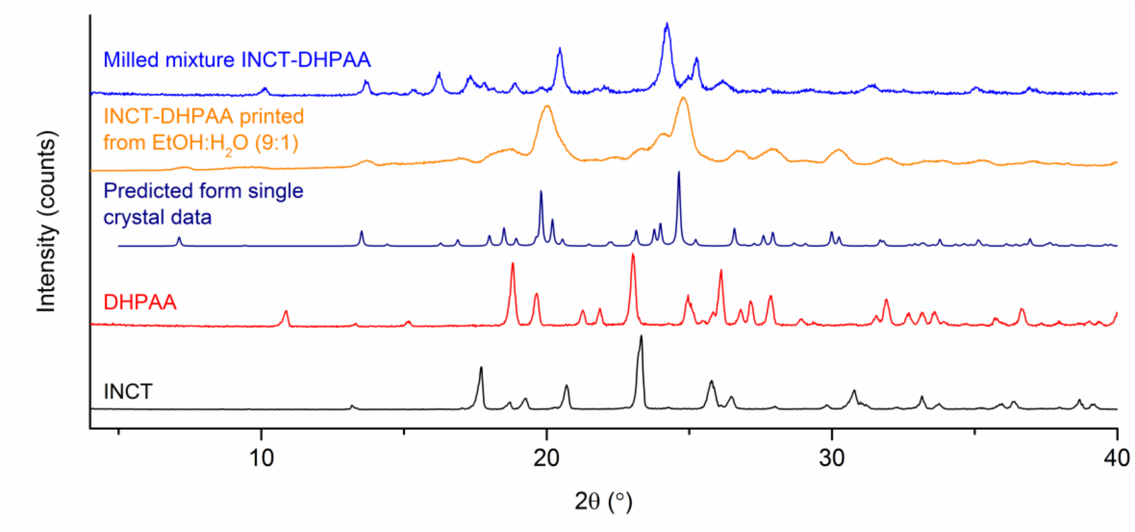

Figure SII.27. XRD patterns collected for INCT and DHPAA, and the materials resulting from inkjet printing or milling.

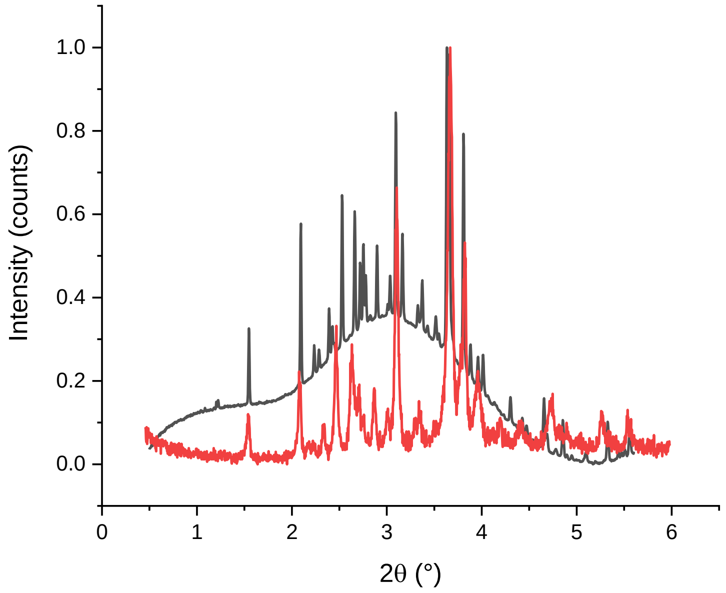

Figure SII.28. A comparison of XRD patterns collected after ball milling a 1:1 mixture of INCT and DHPAA (red), and at 122 °C during DSC-XRD (black).

III. Experimental

Materials: 4-Hydroxyphenylacetic acid, 3,4-dihydroxyphenylacetic acid, 4-hydroxybenzoic acid, L-ascorbic acid, isonicotinamide and absolute ethanol were obtained from Sigma-Aldrich and used as received. Distilled water was used when water was required.

Solvent evaporation: INCT and each of the co-formers were combined in molar ratios of 1:1 for HBA and DPHAA or 2:1 for HPAA, and dissolved in ethanol to the highest concentration possible (i.e. ethanol was added whilst stirring until all solids had dissolved). The solutions were transferred to 96-well plates, which were covered with Parafilm and pierced. Samples were stored at ambient temperature in a fume cupboard, 9 °C in an incubator, 5 °C in a fridge, or 4 °C in an incubator. Selected single crystals were analysed by XRD.

Heat induced co-crystallisation: Binary mixtures of INCT and the four co-formers in the ratios described above were prepared using a vortex mixer. A second set was prepared by grinding each of the components separately in a pestle and mortar for 30 seconds before mixing. Samples were heated by DSC using the instrument and parameters described below.

Thermal inkjet printing: Solutions (3 % w/v in 10 mL of a solvent system comprising EtOH, EtOH:H_2O, 9:1 (v/v), or EtOH:H_2O, 7:3 (v/v)) were loaded into an empty HP 344 Tri-color ink cartridge (foam removed) in an HP Officejet 100 mobile printer. A 10 cm x 10 cm square was printed onto an A4 acetate sheet at 300 dpi (fast draft) with 20 overlays. The printed crystals were removed from the acetate sheet with a spatula for analysis.

Ball milling: Samples of the raw materials at the same molar ratios as above were loaded into the grinding jar of a Retsch MM 200 ball mill and milled at 20 Hz for 15 min.

Characterisation: IR spectroscopy was conducted on a Perkin Elmer Spectrum 100 instrument. All spectra were recorded between 650 and 4000 cm^{-1} with 64 scans at a resolution of 4 cm^{-1}. TGA measurements were performed using a TA Instruments Discovery analyser. Samples were loaded into aluminium cups and heated from ambient to 300 °C at 10 °C min^{-1} with a nitrogen purge of 25 mL min^{-1}. XRD was carried out at room temperature on a Rigaku Miniflex 600 diffractometer fitted with a CuKα source, over the range 3 to 50° with a 0.02° step size and 2° min^{-1} scan rate. DSC measurements were performed on a TA Instruments Q2000 machine. Samples were loaded into Tzero aluminium pans with non-hermetic lids. Calibration was performed with a certified indium standard according to the manufacturer's instructions. Samples were heated to a maximum of 220 °C at 10 °C min^{-1} with a nitrogen purge of 50 mL min^{-1}.

DSC-XRD: DSC Measurements were performed with modified TA 2010 or Q20 instrument (TA Instruments LLC), with holes drilled in the furnace to permit the passage of the X-ray beam as detailed in our previous study.[8] Calibration was performed with a certified indium standard according to the manufacturer's instructions. Samples of all materials (5–20 mg) were held in Tzero aluminium pans and heated at 10 °C min^{-1} from ambient to 220 °C. Experiments were performed on Beamline I12 of the Diamond Light Source using a 0.5 x 0.5 mm beam of monochromated X-rays at 52.4 keV (0.236 Å). A Thales Pixium RF4343 detector, calibrated with a CeO_2 standard, was located 2.4 m away from the sample. Diffraction patterns were recorded every six seconds (data were collected for 4 s with a 2 s pause between collections).

Data analysis: The DAWN Science Workbench was first used to convert the 2D data into 1D diffraction patterns.[9] Contour plots of the raw XRD data were then plotted using OriginPro 2016. Selected patterns were analysed using the Rietveld method implemented within the TOPAS-Academic suite of programmes,[10] in order to obtain realistic values for the unit cell parameters at elevated temperatures. Backgrounds were fitted using a shifted Chebyshev polynomial of the first kind with between 6 and 15 terms. Lattice parameters and peak shape parameters were refined. In cases where more than one phase was present, the peak shapes for each phase were constrained to be the same and the phase fraction was refined. The atom positions were not refined. Atom displacement parameters, U_{iso} were set to be 0.15 Å^2 in each phase. Once starting parameters were obtained, batch refinements were performed on all datasets collected. No zero point was refined as entire diffraction patterns were collected using a 2D area detector.

IV. References

[1] Agilent Technologies Inc, **2014**.

[2] G. M. Sheldrick, *Acta Crystallogr. Sect. A Found. Adv.* **2015**, *71*, 3–8.

[3] G. M. Sheldrick, *Acta Crystallogr. Sect. C Struct. Chem.* **2015**, *71*, 3–8.

[4] O. V. Dolomanov, L. J. Bourhis, R. J. Gildea, J. A. K. Howard, H. Puschmann, *J. Appl. Crystallogr.* **2009**, *42*, 339–341.

[5] I. D. H. Oswald, W. D. S. Motherwell, S. Parsons, *Acta Crystallogr. Sect. E Struct. Reports Online* **2005**, *61*, o3161–o3163.

[6] G. M. Sheldrick, *Acta Crystallogr. Sect. A Found. Crystallogr.* **2008**, *64*, 112–122.

[7] P. Vishweshwar, A. Nangia, V. M. Lynch, *CrystEngComm* **2003**, *5*, 164–168.

[8] A. Clout, A. B. M. Buanz, T. J. Prior, C. Reinhard, Y. Wu, D. O'Hare, G. R. Williams, S. Gaisford, *Anal. Chem.* **2016**, *88*, 10111–10117.

[9] M. Basham, J. Filik, M. T. Wharmby, P. C. Y. Chang, B. El Kassaby, M. Gerring, J. Aishima, K. Levik, B. C. A. Pulford, I. Sikharulidze, et al., *J. Synchrotron Radiat.* **2015**, *22*, 853–858.

[10] A. A. Coelho, J. Evans, I. Evans, A. Kern, S. Parsons, *Powder Diffr.* **2011**, *26*, S22–S25.
